# Marburg and Ebola Virus Infections Elicit a Complex, Muted Inflammatory State in Bats

**DOI:** 10.3390/v15020350

**Published:** 2023-01-26

**Authors:** Anitha D. Jayaprakash, Adam J. Ronk, Abhishek N. Prasad, Michael F. Covington, Kathryn R. Stein, Toni M. Schwarz, Saboor Hekmaty, Karla A. Fenton, Thomas W. Geisbert, Christopher F. Basler, Alexander Bukreyev, Ravi Sachidanandam

**Affiliations:** 1 Girihlet Inc., Oakland, CA 94609, USA; 2 Department of Pathology, the University Texas Medical Branch, Galveston, TX 77555, USA; 3 Galveston National Laboratory, the University of Texas Medical Branch, Galveston, TX 77555, USA; 4 Amaryllis Nucleics, Oakland, CA 94609, USA; 5 Department of Oncological Sciences, Icahn School of Medicine at Mount Sinai, New York, NY 10029, USA; 6 Department of Microbiology, Icahn School of Medicine at Mount Sinai, New York, NY 10029, USA; 7 Department Microbiology & Immunology, the University of Texas Medical Branch, Galveston, TX 77555, USA

**Keywords:** bat, transcriptome, Ebola virus, Marburg virus, inflammation

## Abstract

The Marburg and Ebola filoviruses cause a severe, often fatal, disease in humans and nonhuman primates but have only subclinical effects in bats, including Egyptian rousettes, which are a natural reservoir of Marburg virus. A fundamental question is why these viruses are highly pathogenic in humans but fail to cause disease in bats. To address this question, we infected one cohort of Egyptian rousette bats with Marburg virus and another cohort with Ebola virus and harvested multiple tissues for mRNA expression analysis. While virus transcripts were found primarily in the liver, principal component analysis (PCA) revealed coordinated changes across multiple tissues. Gene signatures in kidney and liver pointed at induction of vasodilation, reduction in coagulation, and changes in the regulation of iron metabolism. Signatures of immune response detected in spleen and liver indicated a robust anti-inflammatory state signified by macrophages in the M2 state and an active T cell response. The evolutionary divergence between bats and humans of many responsive genes might provide a framework for understanding the differing outcomes upon infection by filoviruses. In this study, we outline multiple interconnected pathways that respond to infection by MARV and EBOV, providing insights into the complexity of the mechanisms that enable bats to resist the disease caused by filoviral infections. The results have the potential to aid in the development of new strategies to effectively mitigate and treat the disease caused by these viruses in humans.

## 1. Introduction

The filoviruses Marburg virus (MARV) and Ebola virus (EBOV) cause severe and frequently fatal diseases in humans [1]. The diseases caused by these viruses are characterized by hypotension, multisystem organ failure, sepsis-like symptoms, and disseminated intravascular coagulation (DIC) due to profound immune dysregulation accompanied by a cytokine storm [2]. The aggressive use of a recently approved EBOV vaccine has achieved control of the most recent large outbreak in the Democratic Republic of the Congo [3]. However, once the disease is manifested, medical intervention can prove to be insufficient, despite the recent progress in the development of antivirals and antibody-based treatments [4,5]. Therefore, there is an ongoing need to better understand the pathobiology of these viruses. Remarkably, MARV and EBOV appear to cause no significant disease in their confirmed (MARV) or likely (EBOV) bat reservoirs, suggesting that understanding the molecular mechanisms of this resistance can be used to develop therapies for humans.

MARV has been isolated from the Egyptian rousette bat (ERB) (*Rousettus aegyptiacus)* [6,7,8], and ecological and experimental studies have demonstrated that this species serves as a reservoir for the virus [7,9]. Experimental infections of ERBs with MARV have consistently demonstrated that despite viral replication in multiple tissues, animals develop a mostly subclinical disease. This is characterized by mild pathology, including transient elevation of alanine aminotransferase, elevated lymphocyte and monocyte counts, and some evidence of minimal inflammatory infiltration in the liver [10,11,12,13,14]. Transmission has been demonstrated between co-housed ERBs, and the virus is known to be shed in saliva, urine, and feces [9]. However, ERBs do not appear to develop a severe or chronic infection when exposed to MARV, and instead clear the virus and develop at least temporary immunity, including MARV-specific IgG [15].

Circumstantial evidence, including detection of EBOV RNA and anti-EBOV antibodies, suggests that multiple species of bats are also reservoirs for EBOV [16,17]. However, we are unaware of isolation of infectious EBOV from a wild bat. Although serological surveys have identified antibodies reactive to EBOV antigen in ERBs [18,19,20], experimental infection studies performed to date suggest that this bat species is refractory to infection [21], making it an unlikely candidate reservoir for the virus. 

The ability of bats to tolerate viral infections has been a topic of considerable interest, and several models have been proposed to explain this phenomenon [1,2,3,22,23]. For example, one model posits that bats constitutively express interferons to maintain a basal level of innate immune activity [22], although the universality of this model in bats is uncertain [24,25]. Another model suggests that the resistance of bats to clinical disease is due to a weakened innate immune response, which is attenuated by modifications of some proteins such as the stimulator of interferon genes (STING/TMEM173) [23]. These proposed mechanisms are not necessarily mutually exclusive [26]. While this model can explain viral replication in bats, it is inadequate to explain the eventual viral clearance. Furthermore, the similarity of innate immune responses to filoviruses in bat and human cell lines [27] despite distinct clinical outcomes of the infections in bats versus humans contradicts these theories. Yet another study, based on genomic analysis, hypothesized that evolutionarily divergent genes could explain bat–human differences in responses to viruses [25].

Our study demonstrated that the response of bats to filoviruses involves multiple interrelated processes, and the difference in the responses to infection between bats and humans is driven by the modified response of several genes, including many evolutionarily diverged genes, that are sufficient to change systemic response at the level of pathways. This results in a measured inflammatory response that allows an adaptive response to be mounted to clear the infection and suggests potential therapeutic strategies for controlling the disease caused by filoviruses in humans.

## 2. Materials and Methods

**Viruses.** Recombinant wild-type MARV, strain 371 Bat, was recovered in BHK-21 cells [28] and passaged twice in Vero E6 cells for amplification. Recombinant wild-type EBOV, strain Mayinga, was recovered from the full-length clone and support plasmids in HEK 293 T [29] cells and passaged twice in Vero E6 cells for amplification. Genomes of both viruses were completely sequenced, resulting in the confirmed expected sequences.

**Bat experimental protocol**. All animal procedures were performed in compliance with protocols approved by the Institutional Animal Care and Use Committee at the University of Texas Medical Branch at Galveston.

Adult ERBs were obtained from Wildlife Safari and CJG EXOTICS and quarantined for 30 days under ABSL-2 conditions. Animals were implanted with microchip transponders for animal ID and temperature data collection. To perform MARV and EBOV infections, animals were transferred to the Galveston National Laboratory ABSL-4 facility. Animals were segregated into groups of three. In the infected group, except for one MARV-infected male (bat #3), all bats were female. Male bats are prone to fighting, and as such, should not be cohoused in non-flight caging. Each group was housed in a separate cage for inoculation with the same virus. After acclimation to the facility, animals were anesthetized with isoflurane and infected subcutaneously in the scapular region with 10^4^ PFU (titrated on Vero E6 cells) of MARV or EBOV. Every other day, animals were anesthetized by isoflurane, weighed, temperature was determined via transponder, and 100–150 µL of blood was collected from the propatagial vein. Blood was inactivated in 1 mL of TRIzol reagent (Thermo-Fisher Scientific, Waltham, MA, USA). Samples were then removed from ABSL-4 containment, and RNA was extracted. Droplet digital RT-PCR (ddRT-PCR) with primers specific to the nucleoprotein (NP) gene was used to detect viremia. MARV-infected animals were euthanized on day 7 and EBOV-infected animals were euthanized on day 11 under deep isoflurane sedation via cardiac exsanguination confirmed by bilateral open chest. Tissues were collected (listed in Table S1) and immediately homogenized in an appropriate volume of TRIzol reagent and stored at −80 °C. Then, 1 cubic centimeter (cc) tissue sections were homogenized in minimal essential media (MEM) supplemented with 10% fetal bovine serum and stored at −80 °C. Additional tissue sections were fixed in 10% neutral buffered formalin for histopathology. Tissues and PBMCs were also collected from three uninfected control animals.

**Leukocyte isolation.** Leukocytes were isolated using ACK lysis buffer (Gibco). Ten volumes of lysis buffer were added to whole blood, incubated for 2–3 min, and then neutralized with complete DMEM medium containing 10% FBS. Following neutralization, samples were centrifuged at 250× *g* for 5 min at 4 °C, after which the supernatant was decanted from the pellet. This process was repeated several times per sample until a white pellet of cells free of contaminating red blood cells remained. As density gradient purification was not performed on these samples prior to or after red blood cell lysis, these leukocyte preparations were assumed to contain granulocytes in addition to PBMCs.

**mRNA sequencing.** Total RNA was isolated from bat tissues using Ambion’s RNA isolation and purification kit. For most samples, polyA-tailed mRNA was selected using beads with oligo-deoxythymidine and then fragmented. A few samples with poor RIN (RNA Integrity Number) scores were treated with Ribominus (targeting human ribosomal RNA) to enrich for polyA-tailed mRNA before fragmentation. cDNA was synthesized using random hexamers and ligated with bar-coded adaptors compatible with Illumina’s NextSeq 500 sequencer. A total of 88 samples were sequenced on the NextSeq 500, as 75 base pair single reads. 

**Bat mRNA sequence database.** The extant bat genomes are not complete, and a comprehensive mRNA database does not exist. Thus, for this study, we constructed a custom non-redundant reference bat mRNA sequence database, which is available at the FiloBat website [30]. We started with existing genome annotations [31]. The complications arising from splice variants were avoided by keeping only the longest transcript for each gene. We added missing annotations/sequences (e.g., CYP11B2 and PLG) to our database by assembling reads from our own sequence data. These required custom scripts as there often were not enough reads covering a transcript, which precluded the use of standard assembly tools. The gene sequences were collected from different bat species, so error-free reads might not map perfectly to the transcripts in the database. The database has sequences of 18,443 bat mRNAs and includes MARV and EBOV sequences. The gene sequences were collected from different bat species. 

The genes were identified by homology to mouse and human genes; 16,004 bat genes had high similarity to human or mouse homologues, as defined by BLASTn with default settings identifying matches spanning the length of the mRNA. The set of remaining genes (2439) were labelled as divergent. Of these, 1548 transcripts could be identified by increasing the sensitivity of BLASTn by reducing the word-size from 11 to 9, which is equivalent to matching at the protein level. Of the remaining 891 putative transcripts, homologues for 182 could be identified based on partial homology and domain structure, while the remainder (709 sequences whose names start with UNDEF) belonged to one of four classes, (1) aligned to un-annotated transcripts in the human genome, (2) non-coding RNAs, (3) transcripts unique to bats, or (4) assembly errors. We use capitalizations to represent bat gene symbols, as in the human gene nomenclature.

We selected a subset of genes that had good expression (defined as transcripts-per-million or tpm > 20) in at least one class of liver samples (MARV-infected, EBOV-infected, or uninfected) and responsive in either MARV-infected or EBOV-infected bat livers, which we defined as upregulated (log_2_ ratio > 0.6), or downregulated (log_2_ ratio < −0.6). We were left with 151 genes that are the foundation of our analyses of pathways involved in the response to filoviruses (Tables S3–S8). 

**Expression Analyses.** To determine transcript expression levels, we used Kallisto [32], because this tool uses pseudo-alignments and is relatively more tolerant of errors/variants in reads, which we expect here because the reads and mRNA sequences in the database do not always come from the same species. Kallisto uses a parameter “k” while indexing the database to specify how sensitive it is to matches with smaller k values, leading to more spurious hits. We empirically determined k = 23 to be an appropriate parameter value with which to index the reference mRNA dataset. We used the tpm value as the transcript expression levels to determine changes in expression across samples.

We used viral transcripts to identify infected samples, which has previously helped us to identify and correct errors in annotation in some of the cell line data and also identified a problem with a published dataset [33], where all the naïve (uninfected) samples showed signs of viral infection. Furthermore, to ensure there was no mislabeling of tissue samples from different bats, we used single nucleotide variants in the sequenced reads to confirm that all tissue samples from an individual had the same set of variants. 

Using clustering based on expression profiles and considering individual interferon responsive genes, it was clear that one non-infected control bat liver sample [labeled *cb1* in the FiloBat tool [30]] was reacting to some stimulus (injury or infection) compared to the other two control samples [*cb2* and *cb3* in the FiloBat tool [30]]. Correlations between samples based on gene expressions profiles (Table S9) shows that *cb1* is an outlier and is closer in profile to the MARV-infected samples, suggesting an inflammatory response is present. This animal had an injury, and it is likely that inflammatory processes associated with this were responsible. Since we are interested in the innate response to infections, we had to exclude control sample *cb1* from all analysis, but *cb1* data are available for exploration in the *filobat* tool. Most of our analyses concentrated on liver RNA transcripts since it had the strongest response, and the genes indicated that a variety of cell types were involved in the response, capturing the systemic nature of the response. Liver function impacts a wide range of systems involving inflammation, iron homeostasis, and blood pressure. Other organs, such as kidney and spleen, provide additional support for what is observed in the liver. For some genes, we also used the transcriptional response in kidney (RENIN) and/or spleen (STING) to understand the regulation of pathways (e.g., RENIN is secreted by kidney and regulates the blood pressure system) [34].

**Tools for data exploration and interrogation.** To allow exploration of the data across various samples on a gene-by-gene basis, as well as analysis of viral expression in the samples, we developed a browser-based tool, *FiloBat*, using *Shiny* in R [30]. Samples can also be compared using scatter plots and hierarchical clustering. 

**Statistics.** Large changes in expression profiles were readily detected by comparing averages across replicates, since such changes are less affected by noise; however, subtle changes (less than 2-fold) were difficult to reliably detect due to lack of power in the sample size, and variabilities between samples were mostly not considered. Since only two samples were left in the controls, we could not use the t-test to compute *p*-values for comparisons between the EBOV and control samples. Correlations between the various samples (Table S9) suggest that we could perform comparisons between the MARV and the EBOV (which are broadly similar to the uninfected samples), and over 130 of the 194 genes highlighted in the study have FDR values below 0.25. Many of these (40 genes) would have exceeded this threshold if all the genes in the study were considered. The full table of genes with the *p*-values and FDR is available on the companion website [30]. 

**Pathway analyses.** A fundamental assumption underlying our study is that bats are mammals that possess innate and adaptive responses to infections that roughly mirror those seen in humans. The data from comparative filovirus infections in human and bat cell lines support this assumption [27]. To identify pathways of interest from particular genes, we used GO/pathways annotations of the human counterparts [35] and grouped them into functions that provided themes in the dataset. Using these themes, we identified other differentially expressed genes sharing these themes, identified by the GO annotations for human and mouse genes. This allowed us to build a picture of the pathways triggered by filovirus infections and delineate the ways in which the systemic bat responses differs from those seen in humans. 

## 3. Results

### 3.1. Inoculation of Bats with MARV and EBOV Results in Detectable viral Replication in Some Organs

**Figure 1:**
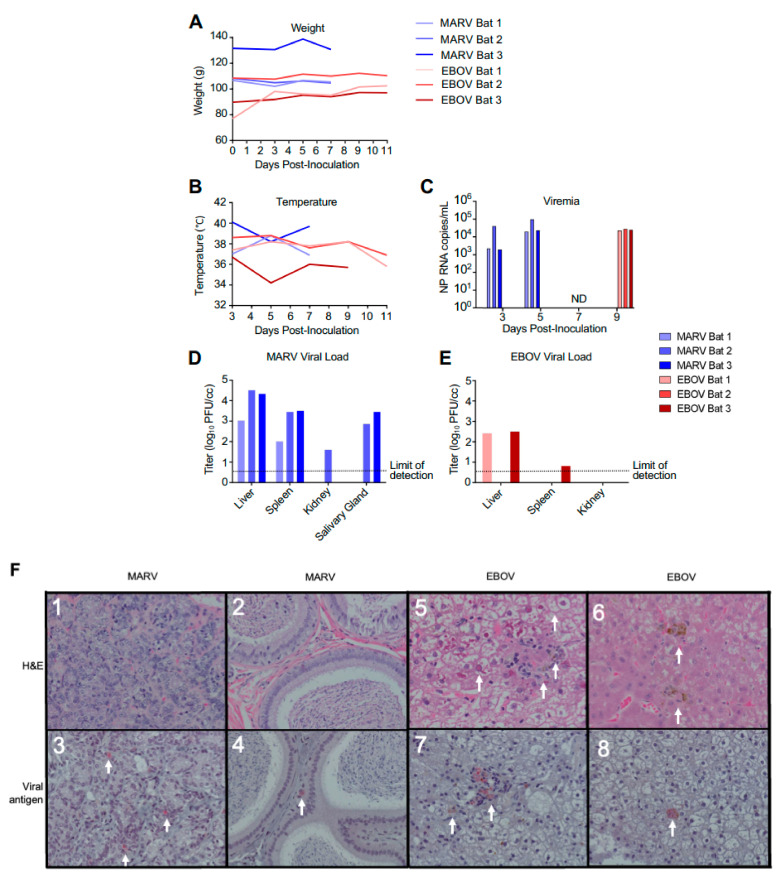
Infection of bats with filoviruses MARV and EBOV. (**A**–**C**). Time course after infection for weight (**A**), temperature (**B**), and viral RNA specific for the NP gene in total RNA extracted from whole blood (**C**). Animals were euthanized 48 h after first viremic time point. ND, not detected for EBOV (the MARV-infected bats were euthanized at this time point). (**D**,**E**). Tissue viral loads on day 7 for EBOV (**D**) and day 11 for MARV (**E**) determined by plaque assay. Note that transponders in MARV-infected bat 2 and EBOV-infected bat 1 on 11 dpi failed. (**F**). Histopathology and viral antigen in filovirus infected organs showing: (**F1**) Lack of significant histopathological lesions in mammary tissue from MARV-infected bat 1, (**F2**) Lack of significant histopathological lesions within the interstitium of the epididymis from MARV-infected bat 3, (**F3**) IHC detection of MARV antigen within the interstitium of the glandular structures of the mammary gland from MARV-infected bat 1 (red pigment, arrows), (**F4**) IHC detection of MARV antigen within the interstitium of the epididymis from MARV-infected bat 3 (red pigment, arrows), (**F5**) EBOV-infected bat 1 liver with marked histopathological changes, including cytoplasmic and nuclear inclusions (arrows), (**F6**) EBOV-infected bat 2 liver displaying a less dramatic presentation compared to bat 1 (arrows), (**F7**) IHC detection of EBOV antigen in the liver of EBOV-infected bat 1 (arrows), (**F8**) IHC detection of EBOV antigen in EBOV-infected bat 1 liver (an arrow).

Nine ERBs were either inoculated subcutaneously with 10^4^ PFU of MARV or EBOV or were left uninfected (three in each group). Following inoculation, animals were observed at least daily and bled every other day. As the goal of the study was to investigate changes in gene expression in the early phase of the viral clearance, the MARV-infected animals were euthanized on day 7, which is approximately 2 days after the expected peak of the viral replication based on a previously published study [14]. Previous experimental infections of ERBs with EBOV resulted in detection of low levels of viral RNA (but not live virus) in the blood and some organs on days 3–16 after infection [14], suggesting a possible slow replication of the virus. Because of that, a later time point—day 11—was selected for euthanasia of EBOV-infected ERBs. As expected, bats inoculated with MARV or EBOV showed no apparent clinical signs of disease or changes in behavior, with no significant effect on body weight and temperature (Figure 1A,B). MARV or EBOV RNA was detected by ddRT-PCR in the blood of infected bats (Figure 1C). MARV was detected by plaque assay in livers and spleens of all inoculated animals and in the salivary glands of two animals and kidneys of one animal (Figure 1D). By contrast, EBOV was detected in the livers of two inoculated animals and could not be reliably detected elsewhere (Figure 1E). 

No pathology was observed in sections of mammary tissue or in the testes of MARV-inoculated animals (Figure 1F1,2), that is consistent with previous reports [14]. However, immunohistochemistry analysis demonstrated MARV antigen in both tissues, though this was focal in nature (Figure 1F3,4), despite the absence of histopathological lesions in these organs. Two of the three EBOV-inoculated animals presented histopathological lesions in the liver, consisting of pigmented and unpigmented infiltrates of aggregated mononuclear cells compressing adjacent tissue structures, and eosinophilic nuclear and cytoplasmic inclusions (Figure 1F5,6), the changes consistent with previous reports [19,21]. Immunohistochemical analysis demonstrated EBOV antigen in the liver of one animal, but very few foci were found (Figure 1F7,8), suggesting limited viral replication. Overall, the histological studies demonstrated moderate amounts of MARV antigen and low amounts of EBOV antigen, that is consistent with the virus load data generated by plaque titration and ddRT-PCR, and very limited pathological changes.

### 3.2. MARV and EBOV Infection Affects the Transcriptome of Multiple Organs

To examine the transcriptional response to filovirus infections, we performed deep sequencing of mRNA from liver, spleen, kidneys, lungs, and large intestine collected from filovirus inoculated and uninfected bats (Methods, Table S1). We focused on analysis of the transcriptional response in liver, spleen, and kidneys as these organs are classic targets of filovirus infections. Consistent with prior reports that liver is the primary target of MARV [36], and with our findings (Figure 1), MARV transcripts were the most abundant in liver (79 transcripts-per-million or tpm) and also present in spleen (56 tpm), intestine (10 tpm), and lungs (2 tpm), but not present in kidneys (Table S2). EBOV transcripts were detected at very low levels (<1 tpm) in the livers of inoculated bats and were not detectable in other tissues. 

**Figure 2:**
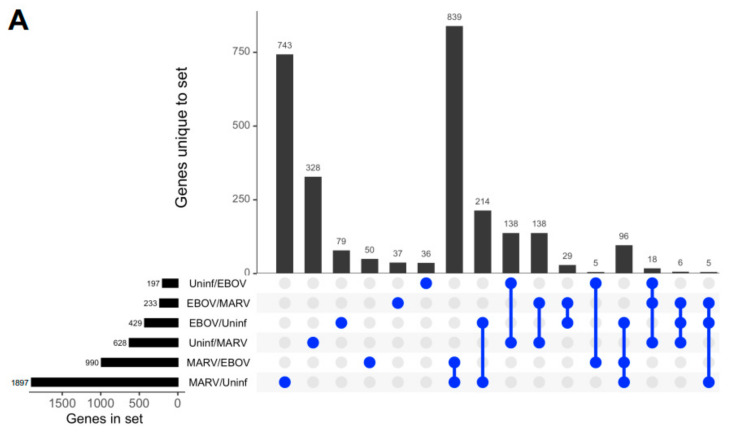

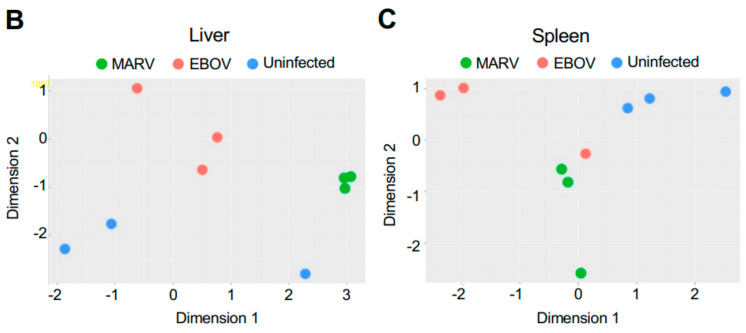
Changes in gene expression patterns in bats after infections with MARV and EBOV. (**A**). An UpSet plot of the gene expression data in the livers of bats infected with MARV, EBOV, or uninfected. Responsive genes are placed into six groups (MARV/Uninf, MARV/EBOV, EBOV/Uninf, EBOV/MARV, Uninf/MARV, and Uninf/EBOV) listed in the lower left panel. Each group consists of genes whose expression is increased at least 2-fold versus the comparator, e.g., EBOV/Uninf comprises genes upregulated in EBOV-infected bats compared to the uninfected bats, while Uninf/EBOV comprises genes downregulated in EBOV-infected bats compared to the uninfected bats. A gene can belong to multiple groups, as indicated by the blue dots. The first vertical bar in the graph represents the 743 genes that are unique to the MARV/Uninf set, while the last bar represents the 5 genes that occur only in the combination of 3 sets, EBOV/MARV, EBOV/Uninf and MARV/Uninf. In the lower left bar plot, the first horizontal bar represents the 197 genes that belong to Uninf/EBOV, which is a sum of the values for the four combinations which include the Uninf/EBOV comparison (36 + 138 + 5 + 18). (**B**,**C**). Multidimensional scaling (MDS) plots of the merged gene expression data in livers (**B**) and the spleens (**C**) of bats infected with MARV, EBOV, or uninfected. One uninfected bat in panel B is an outlier based on comparison to the two other uninfected bats, which is also seen in the correlations shown in Table S9. The difference for this bat may be explained by its reaction to some stimulus, either an infection or an injury; the animal has been excluded from the downstream analysis. Virus-specific signatures were also detected in kidneys (Figure S1), implying the response to filovirus infections extends to the whole animal.

Remarkably, although the highest levels of viral transcripts were detected in the liver, gene expression patterns were altered in all three tissues subjected to transcriptome analysis, liver, spleen, and kidneys, involving thousands of genes, suggesting a systemic response (Figure 2 and Figure S1). The changes were higher in the livers of bats infected with MARV (1897 genes) than EBOV (429 genes) (Figure 2A), which can be explained by the much more abundant replication of MARV (Figure 1). The sets of genes affected by the two infections were not completely identical, suggesting some responses are virus-specific (Figure 2A and Figure S1). The differences in the patterns of gene expression could arise from different mechanisms of interferon antagonism, which is also mediated by different proteins: VP35 and VP40 in MARV and VP35 and VP24 in EBOV (reviewed in [4]. Multidimensional scaling (MDS) plots of gene expression in livers (Figure 2B), spleens (Figure 2C), and kidneys (Figure S1) of MARV-infected, EBOV-infected, and uninfected bats showed a clear separation due to virus-specific signatures, except for the spleen from one EBOV-infected animal, which was close to the spleens of two MARV-infected animals. The detection of these virus-specific signatures in multiple organs implies the virus-specific responses extend to the whole animal.

### 3.3. Understanding the Response to Filovirus Infection Using the Pathways Framework 

**Figure 3:**
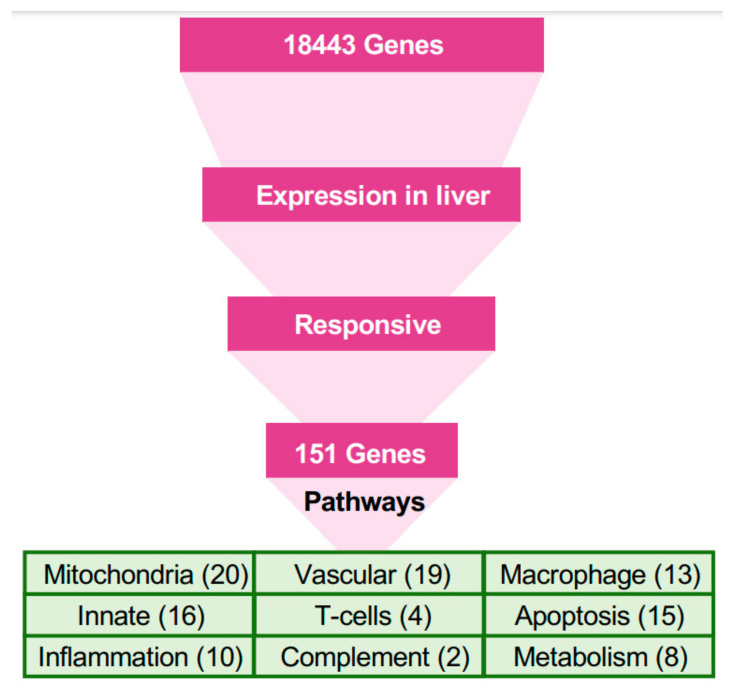
A pathway-based approach for understanding the response to filovirus infections. The approach used in this study was based on identification of pathways relevant to the bat’s resilience to filoviral infections, under the assumption that homologous genes perform similar functions in bats and humans. Since the liver is the locus of viral replication (viral transcripts are mostly found in the mRNA from livers), genes that were highly expressed in livers and responsive to the infection in bats were first identified. The genes were mapped to pathways, and other genes in the pathways were evaluated to identify the systemic response to filovirus infections in bats and to identify key differences from the responses in humans. Blood pressure, coagulation, and iron homeostasis pathways were altered most prominently. The analysis demonstrated changes in glycolysis, which is controlled by hypoxia, which shift the balance of macrophage activation from the M1 (proinflammatory) to the M2 (anti-inflammatory) state. These changes create a pro-inflammatory state that modulates the response and allows the adaptive immune system to clear the infection early, and anti-inflammatory response late. The identified pathways demonstrated incomplete activation of the complement system, likely compromising the antibody response, but strong activation of T cell response, which is likely to play a major role in clearing the infection. The identified pathways are interconnected, leading to the coordinated changes shown in Figure 4 and connections delineated in Figure 5.

The stark difference in the outcomes of filovirus infections in bats versus humans, despite substantial parts of the response being similar, suggests a few pathways respond differently in bats, compared to humans. We selected genes that responded differently in bats and identified their putative membership in pathways, and then used all genes in these pathways for further analysis (Figure 3, Methods).

The major benefit of limiting the genes to the responsive genes in liver is that we ameliorate the multiple-testing problem. For example, 130 liver-specific genes were differentially responsive upon comparing the response to MARV and EBOV infections with a false discovery rate (FDR) < 0.25. In contrast, starting from the full list of bat genes in the genome would yield only 90 genes with FDR < 0.25, due to the false positives from the larger set of background genes reducing the signal (table shown in accompanying website [30].

Only 151 genes (Figure 3) were responsive to either MARV or EBOV in the liver (Figure S2). These 151 genes were the basis of the first step of pathway analysis (Tables S3–S8). The most abundant group in this set comprised genes related to mitochondria (20 genes), followed by genes involved in the vascular system (19), innate immunity (16), tissue regeneration and apoptosis (15), macrophages (13), inflammation (10), metabolism and fatty-acid oxidation (8), T cells (4), complement system (2), digestion (5), and toxin processing (3). 

Based on this preliminary list of pathways, we focused on the *entire* transcriptomes of the following systems: (1) innate immune system (Figure S3) which includes the interferon stimulated genes (Figure S4), most of which are conserved between bats and humans, phagocytosis by macrophages, and natural killer cells and the complement system; (2) inflammatory response, including acute phase proteins, macrophages functions involving metabolism, fatty-acid oxidation, mitochondrial abundance and function, and tissue regeneration and apoptosis; and (3) blood-related physiological systems, involving the regulation of blood pressure, coagulation, and iron homeostasis.

### 3.4. Effects of Filovirus Infections on the Transcriptional Response

**Figure 4:**
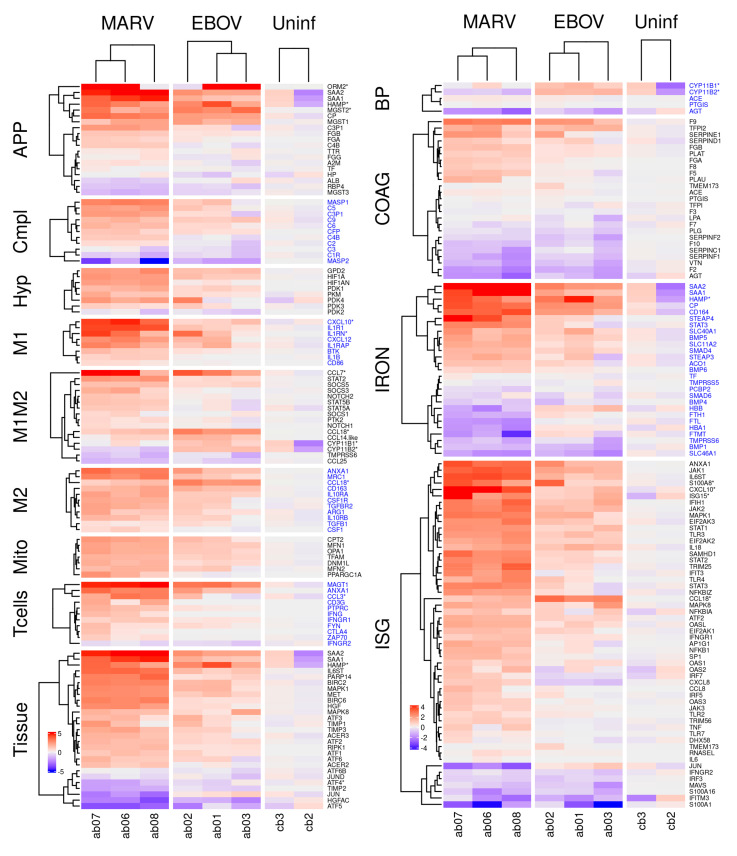
Differential expression of genes belonging to the indicated pathways by MARV and EBOV infections in livers of bats. The columns show differentially expressed genes from livers of three MARV-infected bats, three EBOV-infected bats and two uninfected bats. The values are log2 of the fpm values, with the mean values from uninfected animals subtracted. APP: acute phase response proteins; Compl: complement; Hyp: hypoxia related genes; M1 and M2: genes specific to M1 or M2 macrophages, respectively; M1M2: genes common to M1 and M2 macrophages; Mito: genes expressed in mitochondria; Tcells: genes expressed in T cells; Tissue: genes involved in tissue regeneration and apoptosis; BP: genes involved in regulation of blood pressure; ISG: interferon stimulated genes; IRON: genes involved in iron homeostasis; COAG: genes involved in coagulation. A * after a gene name indicates that the bat version is divergent from its human counterpart. Alternate blocks of gene names are colored black/blue to allow easy visual distinction of the blocks. Figure S2A,B show corresponding figures for kidneys and spleens.

Analysis of the transcriptome in livers (Figure 4), kidneys (Figure S2A), and spleens (Figure S2B) demonstrated significant changes in expression of genes associated with the infections, often coordinated across organs (such as the blood pressure regulation genes between liver and kidney). Sometimes the changes of expression detected in livers were less pronounced or not observed in kidneys and spleens, consistent with the livers exhibiting the highest viral replication. In some pathways, such as the complement pathway, the MARV response was stronger, consistent with the higher replication of MARV in the infected bats.

*MARV and EBOV infection induce initial inflammation, evidenced primarily by an acute phase response.* Acute phase proteins (APP) are produced by hepatocytes in the liver in response to inflammatory cytokines, such as interleukin (IL)-1, IL-6, and tumor necrosis factor α (TNFα), and are an important part of the innate immune response [37,38,39]. Upon inflammation, the concentration of positive APPs, including serum amyloid A1 (SAA1) and serum amyloid A2 (SAA2), increase dramatically (> 10-fold) in the serum [40], while the concentration of negative APPs, including transferrin (TF) and albumin (ALB), decreases [41].

We found that MARV, and to a lesser extent EBOV, infection induced an APP response in liver Figure 4), spleen (Figure S2B), and kidney (Figure S2A), with the largest changes in APP expression (>10-fold) observed in the liver (Table 1, Figure 4). SAA1 and SAA2 expression increased to a similar degree in all tissues, including tissues in which the viruses were not detected (Figure 4 and Figure S2A,B). At the same time, we detected no expression of C-reactive protein (CRP), an APP used as a marker for inflammation/acute-phase-response in humans (Table 1), likely because it is not expressed in bats. We draw this conclusion in part because we were also unable to identify evidence of a CRP response in an analysis of public mRNA-seq data from infected samples from various species of bats. Consistent with the induction of SAA1 and SAA2, we also detected induction of other markers of inflammation including orosomucoid 2 (ORM2: Table 1, Figure 4 APP, Figure S2A,B APP), ceruloplasmin (CP: Table 1, Figure 4 APP, Figure S2A,B APP), hepcidin (HAMP: Table 1, Figure 4 APP) and the microsomal glutathione S-transferases (MGST1, MGST2: Table 1, Figure 4 APP, Figure S2A APP) [42]. Some of them were more responsive in the EBOV-infected bats, e.g., HAMP, ORM2, and MGST2.

*Expression of key components of the classical complement pathway is inhibited by filovirus infection.* Several key genes associated with the classical complement pathway, which recognizes antibodies bound to antigens, were upregulated by filovirus infection (mostly upon MARV infection, to a lesser extent with EBOV), including complement component 3 precursor pseudogene (C3P1: Figure 4 Cmpl, Figure S5A), complement C4B (C4B: Figure 4 Cmpl, Figure S5A), complement C5 (C5: Figure 4 Cmpl, Figure S5A), complement C9 (C9: Figure 4 Cmpl, Figure S5A), complement C6 (C6: Figure 4 Cmpl, Figure S5A), and mannan binding lectin serine peptidase 1 (MASP1 Figure 4 Cmpl, Figure S5A), while others were downregulated or not expressed, including complement C1r (C1R Figure 4 Cmpl, Figure S5A), complement C3 (C3 Figure 4 Cmpl, Figure S5A), complement C8 gamma chain (C8G), and mannan binding lectin serine peptidase 2 (MASP2:Figure 4 Cmpl, Figure S5A). These observations indicated that the complement pathway is affected by filovirus infection and suggests that some protective mechanisms involving the complement system such as antibody-dependent complement deposition may be compromised. Of note, most of the genes implicated in this pathway are non-divergent between bats and humans. 

*Infected bats exhibit transcriptional signatures of T cell activity*. CD4^+^ T cells recognize peptides presented on MHC class II molecules expressed by antigen-presenting cells (APCs), while CD8^+^ T cells recognize peptides presented by MHC class I molecules, expressed by all nucleated cells [43]. CD8^+^ T cells are cytotoxic and can kill virus-infected cells. Multiple genes expressed only by CD8^+^ T cells, including C-C motif chemokine ligand 3 (CCL3: Figure 4 T cells, Figure S2A,B T cells, Figure S5B, Table S3A), annexin A1 (ANAX1: Figure 4 T cells, Figure S2A,B T cells, Figure S5B), T cell immunoglobulin and mucin domain containing 4 (TIMD4: Table S3A) and magnesium transporter 1 (MAGT1: Figure 4 T cells, Figure S5B), were upregulated in the liver by filovirus infection indicating an induction of T cell response. 

*MARV and EBOV infections affect blood related physiological systems*. The key physiological systems connected to the immune response and inflammation are iron homeostasis, blood pressure, and blood coagulation. We found that expression of genes involved in regulation of all three of these systems was affected by MARV and EBOV infections, as detailed below.

*Genes regulating iron homeostasis*. The absorption and availability of iron, an essential component of heme needed for oxygen transport, is tightly regulated [44]. In humans, most iron in the body is located in hemoglobin (66%) and myoglobin (10%) [45], while the remainder is stored mostly in macrophages in the liver, which take up iron through the CD163 receptor. Iron is exported from macrophages and absorbed from food [46] through ferroportin (SLC40A1/FPN1). 

MARV and EBOV infections changed the expression of multiple genes involved in iron homeostasis. Hepcidin (HAMP: Figure 4 IRON, Figure S2A,B IRON, Figures S5C and S6), which controls iron homeostasis by binding ferroportin [47,48], thereby causing its degradation as well as blocking the export of iron, was induced in infected livers (Figure 4 and Figure S6, Table 1). Similarly, ceruloplasmin (CP) (an APP, Table 1, Figure 4 IRON, Figure S2A,B IRON, Figure S6), which enables the formation of the transferrin-iron complex and is also involved in processing copper [49], was also induced. In the cytosol, iron is bound to ferritin (comprised of a heavy chain, FTH1 and a light chain FTL: Figure 4 IRON, Figure S2A,B IRON, Figure S6), synthesized by cells in response to increased iron [48]. In mitochondria, iron is bound to the mitochondrial ferritin (FTMT: Figure 4 IRON, Figure S2A,B IRON, Figure S6) [50]. Both FTH1 and FTMT were downregulated in MARV-infected but not EBOV-infected bats (Figure 4 and Figure S6); the difference can be explained by higher MARV replication relative to EBOV. Furthermore, MARV infection was associated with lowered hemoglobin (HBB: Figure 4 IRON, Figure S2A IRON, Figure S6) expression, suggesting impairment of red blood cell production. Consistent with this conclusion, CD164 (Figure 4 IRON, Figure S2A,B IRON, Figure S6), which suppresses hematopoietic cell proliferation, was also upregulated by MARV, and to a lesser degree, EBOV infection (Figure 4, Figures S2 and S6). Thus, hematopoiesis may be impaired in MARV-infected ERBs, but not in EBOV-infected ERBs, the difference consistent with the levels of viral replication for the respective viruses in ERBs. Furthermore, as the higher levels of HAMP in EBOV-infected bats do not result in lower levels of FTH1/FTMT, the regulation of iron by HAMP in bats is likely to be diverged from the homologous process in humans [47,48].

*Genes regulating blood pressure*. The primary means of blood pressure regulation is renal expression of renin, which converts angiotensinogen (AGT) to angiotensin I. Angiotensin converting enzyme (ACE) converts angiotensin I to angiotensin II, which constricts blood vessels to increase blood pressure [51]. Both MARV and EBOV infections downregulated AGT (Figure 4 BP, Figure S2A,B BP, Figure S7), resulting in depletion of the substrate for ACE (Figure 4 BP, Figure S2B BP), limiting the potential for blood pressure to increase even with upregulation of ACE. Another mechanism regulating blood pressure involves cytochrome P450 family 11 subfamily B member 2 (CYP11B2), which increases aldosterone levels that increases blood volume and, consequently, blood pressure [52]. CYP11B2 was found to be downregulated in MARV-infected bats in this study (Figure 4 BP, Figure S7), further suggesting the possibility that low blood pressure is a response to MARV infection. In the case of EBOV, CYP11B2 was not downregulated (Figure 4 BP, Figure S2B BP, Figure S7), possibly due to the low level of viral replication. However, blood pressure was not measured in the bats in this study.

*Genes regulating blood coagulation*. Mechanisms that control blood pressure also impact coagulation and vice versa; increasing coagulation leads to higher blood pressure through constriction of blood vessels. For example, fibrinogen B (FGB) is cleaved by thrombin to generate fibrin (which forms the clots), and cleavage products of FGB promote vasoconstriction. The complement pathway also impacts coagulation [53]. 

Coagulation or clotting is a complex process involving a cascade of activation reactions that finally results in thrombin forming clots (Figure S8). Since many of the gene products involved need to be activated, information on the coagulation state is not readily observed from mRNA abundance, which can at most indicate the protein abundance, not their states of activation. Of the genes in the coagulation pathway, MARV and EBOV infections upregulated one set of genes: coagulation factor IX (F9: Figure 4. COAG, Figure S9B), tissue factor pathway inhibitor 2 (TFPI2: Figure 4 COAG, Figure S9B), serpin family E member 1 (SERPINE1: Figure 4 COAG, Figure S9B), SERPIND1 (Figure 4 COAG, Figure S9B), fibrinogen beta chain (FGB: Table 1, Figure 4 COAG, Figure S2B COAG, Figure S9B), plasminogen activator tissue type (PLAT: Figure 4 COAG, Figure S2B COAG, Figure S9B), and downregulated another set: thrombin (F2: Figure 4 COAG, S2A-B COAG, S9B), vitronectin (VTN: Figure 4 COAG, Figure S2B COAG, Figure S9B), SERPINF1 (Figure 4 COAG, Figure S9), SERPINC1 (Figure 4 COAG, Figure S2A,B COAG, Figure S9B), coagulation factor X (F10: Figure 4 COAG, Figure S9B), and plasminogen (PLG: Figure 4 COAG, Figure S2A,B COAG, Figure S9B) (Figure 4, Figures S8 and S9). There were other genes which were not strongly up- or downregulated upon the infections.

In the coagulation cascade (Figure S8), tissue factor III (encoded by the F3 gene) activates coagulation factor VII (encoded by the F7 gene), which then activates several factors in the cascade, eventually activating coagulation factor X (encoded by the F10 gene) which in turn activates coagulation factor II (encoded by the F2 gene) to form thrombin, which enables fibrin synthesis, leading to clot formation. Thrombin is also involved in positive feedback enabling more thrombin to be created. An opposing process involves the conversion of plasminogen to plasmin (facilitated by the plasminogen activators uPA and tPA), which promotes fibrinolysis [54], leading to dissolution of clots. SERPINE1 inhibits this process by inhibiting the activity of uPA and tPA (Figure S8). 

F3 was not abundant in any sample, and F2 was downregulated in response to MARV and EBOV infections, suggesting thrombin production was curtailed; SERPINE1 was upregulated, which can only prevent dissolution of clots, but does not enhance coagulation directly (Figure 4 COAG, Figure S2A,B COAG, Figure S9). Angiotensin II, which increases blood pressure, also increases thrombin formation and impairs fibrinolysis [55]. AGT, the precursor of angiotensin II, was downregulated by MARV and EBOV infections, which is expected to lower angiotensin II levels and concomitantly decrease blood pressure and coagulation (Figure 4 BP, Figure S2A BP, Figure S7). Interestingly, a link between some acute human viral infections and hypotension has been established, including for COVID-19 [56]. Thus, we conclude that filovirus infections of bats lead to reduced coagulation. Together, these events are likely to reduce the effects of inflammation on the vascular system.

*MARV and EBOV infection leads to an early transition from proinflammatory M1 to anti-inflammatory M2-dominated populations of macrophages*. Macrophages, which phagocytize microbes and damaged hosts cells, are an important early target for filoviruses [36]. Macrophages can be in a continuum between the M1 state, an inflammatory state enabling apoptosis, and the M2 state, an anti-inflammatory state assisting tissue regeneration (Figure S5C). A key difference between the M1 and M2 states lies in their metabolism; the M1 state is characterized by hypoxia and glycolysis metabolism [57], while the M2 state is characterized by fatty acid metabolism and elevated mitochondrial activity [58].

We have found that key markers of the M1 state were upregulated in livers of infected bats and more so in MARV infected animals (Figure 4). These markers included interferon regulatory factor 5 (IRF5: Figure 4 ISG), nuclear factor kappa B subunit 1 (NFKB1: Figure 4 ISG), adaptor related protein complex 1 subunit gamma 1 (AP1G1: Figure 4 ISG), signal transducer and activator of transcription 1 (STAT1: Figure 4 ISG), and suppressor of cytokine signaling 3 (SOCS3: Figure 4 M1M2, S10, S11A). Likewise, hypoxia inducible factor 1 subunit alpha (HIF1A) [59,60,61], which promotes mitophagy and glycolysis metabolism to induce M1 polarization, was also upregulated in infected livers, again more so in MARV infection (Figure 4 Hyp, Figure S11B). Pyruvate kinase M1/2 (PKM1/2), which activate HIF1A, and pyruvate dehydrogenase kinase 1 (PDK), which enhances M2 polarization in response to hypoxia [62], were also upregulated (Figure 4 Hyp, S11B. Again, upregulation occurred to a greater degree in MARV than EBOV (Figure 4 Hyp, Figures S10 and S11B).

Markers of the M2 state, such as mannose receptor C-Type 1 (MRC1), arginase 1(ARG1), IL-10 and transforming growth factor beta 1 (TGFB1) [63], were highly expressed in livers of bats infected with both viruses (Figure 4 M2, Figures S10 and S11E), suggesting that M2 macrophages were also present. Several genes related to fatty acid oxidation in M2 macrophages were upregulated by MARV and EBOV infections (Tables S3, S5 and S7). Particularly, we observed upregulation of carnitine palmitoyl transferase 2 (CPT2: Figure 4 Mito), a gene associated with fatty acid transport; the effect was greater in MARV infection. Infected bats also exhibited upregulation of multiple markers of mitochondria abundance, another characteristic of M2 macrophages (Figure S11C). These included transcription factor A mitochondrial (TFAM: Figure 4 Mito, Figure S11C), OPA1 mitochondrial dynamin like GTPase (OPA1: Figure 4 Mito, Figure S11C), mitofusin (MFN1/2: Figure 4 Mito, Figure S11C), and dynamin 1 like (DNM1L: Figure 4 Mito, Figure S11C). Further, upregulated upon MARV infection are genes involved in mitochondrial biogenesis [64], hepatocyte growth factor/tyrosine kinase MET (HGF/MET: Figure S5) and peroxisome proliferator-activated receptor gamma coactivator 1 alpha (PPARGC1A: Figure 4 Mito).

Prolonged M1 activity can be harmful to tissues due to their induction of inflammation and apoptosis. This activity is modulated by a negative feedback system that shifts macrophages from the M1 state to the M2 state [65,66], controlling inflammation during infection and facilitating the transition to tissue repair and regeneration [67,68]. In our data, the transcriptomes of the MARV-infected liver samples exhibited markers for M1 and M2 macrophages, while in the EBOV-infected liver samples, markers for M2 macrophages dominated. These data suggest that the presence of both M1 and M2 macrophages upon MARV infection might be indicative of the greater magnitude and duration of its replication, and the presence of M2 macrophages upon EBOV infection indicates at a conversion from M1 to M2 state during or after the virus clearance.

Consistent with this conversion, we observed changes in cellular energy metabolism that are associated with the M1 to M2 transition. The mitochondrial glycerol-3-phosphate dehydrogenase (GPD2), identified as a contributor to the shift in core macrophage metabolism associated with the M1 to M2 transition during infection [69], was found to be upregulated by filovirus infections (Figure 4 Hyp, Figure S11B). Filovirus infections of bats resulted in upregulation of hypoxia inducible factor 1 subunit alpha inhibitor (HIF1AN: Figure 4 Hyp, Figure S11B), the inhibitor of HIF1A (Figure 4 Hyp, Figure S11B); inactivating HIF1A also promotes M2 polarization [70], suggesting a mechanism of M2 polarization in filovirus infected bats. Interestingly, in MARV-infected bats, which demonstrated a mixed M1-M2 response, we observed downregulation of ferritin (FTH1/FTL: Figure 4 IRON), which is synthesized in response to iron, and HBB (a reflection of hemoglobin levels: Figure 4 IRON), and upregulation of HAMP (Figure 4 APP, Tissue, IRON), which reduces the levels of iron. In EBOV-infected animals, which demonstrated a more pronounced M2 response (Figure S11D,E), no downregulation of FTH1/FTL (Figure 4 IRON), a small upregulation of HBB (Figure 4 IRON) and a stronger upregulation of HAMP (Figure 4 APP, Tissue, IRON) was detected. These data are consistent with the data that an increased availability of iron promotes the M1 to M2 polarization shift [71]. These lines of evidence further support our findings of a polarization bias toward M2 during filovirus infections.

## 4. Discussion

Over the past few years, multiple high-impact pathogens associated with bats have emerged or re-emerged, including, but not limited to, MARV, EBOV, severe acute respiratory coronavirus (SARS-CoV), SARS-CoV-2, Middle East respiratory coronavirus (MERS-CoV), Nipah virus, and Hendra virus. As a result, the role of bats as reservoirs for a diverse array of viruses and their ability to tolerate viral infections that cause severe disease in humans has become a topic of considerable interest. Several hypotheses, mostly centered on the innate immune system, have been proposed to explain this unique aspect of bat biology. In these hypotheses, various aspects of bat innate immunity are either more or less potent than their human counterparts. One of these hypotheses posits that some bat species constitutively express interferons, leading to a basal level of innate immune activation [22,24,25]. However, in humans, persistent interferon expression leads to lowered resistance to infections due to dysregulation of iron homeostasis [72] and is associated with various pathologies [73]. Patients with trisomy have elevated constitutive expression of interferons and exhibit weaker immune response in COVID-19 [74,75]. These pathologies and prior work with filoviruses demonstrating that the innate response in bat cells is robust, and similar to that observed in human cell lines [27], suggesting that interferons in bats are induced similarly to that observed in humans. Thus, the hypothesis of constitutive high expression of interferons in bats is inconsistent with our data. Another hypothesis suggests that components of the innate immune response, e.g., stimulator of interferon response cGAMP interactor 1 (STING/TMEM173), are less effective in bats, allowing viruses to survive in the host [23]. This mechanism cannot explain the virus clearance, although it might explain the lack of symptoms during the infections in bats. The eventual clearance of filoviral infections by bats suggests a more complex process involving both the innate and adaptive immune systems. 

In this study, all MARV-inoculated bats were productively infected, and our virology and histopathology data in MARV-infected bats are consistent with previous reports, including viral replication in the mammary glands and testes [14]. We note that that persistent MARV infection was detected in testes of non-human primate survivors [76]. Unexpectedly, evidence of productive, while limited, infection was identified by ddRT-PCR in all three EBOV-infected bats and by plaque assay in two of the three EBOV-inoculated animals, although the detection of virus in the livers by plaque assay and immunohistochemistry identified only a small number of foci in the liver of one animal (Figure 1C, E). These data contrast the prior reports [14,77] and suggests that ERBs may not be truly refractory to EBOV infection. However, given the low titers detected, and the limited nature of the observed immunostaining, it remains to be determined whether the virus can be maintained in this bat species in nature.

**Figure 5:**
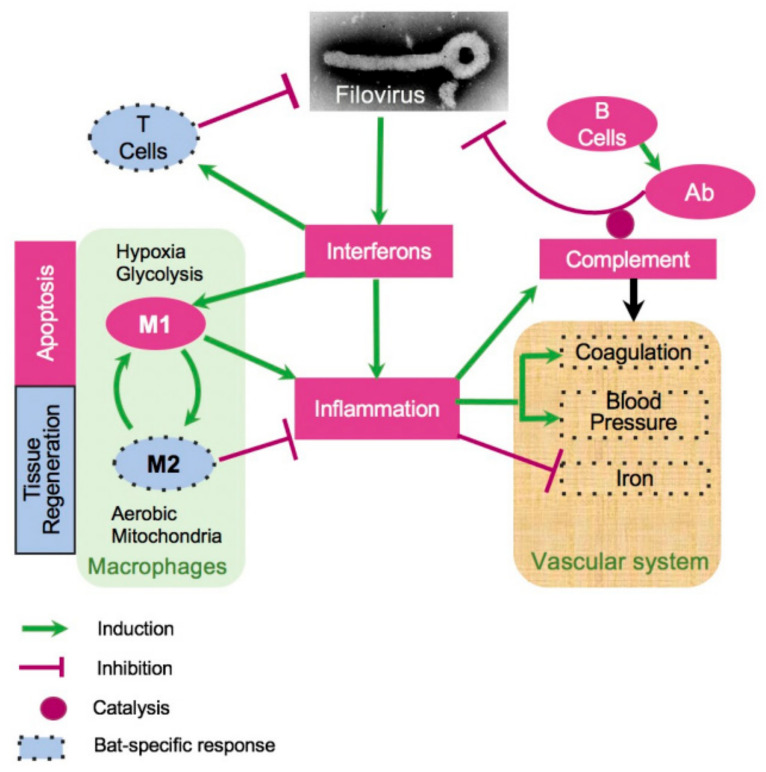
A model of bat response to filovirus infections. Interferon stimulated genes (ISG, Figure 4, Figures S2 and S3) cause inflammation, which triggers the acute phase response (APR, Table 1, Figure 4), leading to a cascade of reactions affecting regulation of HAMP (iron, Figure 4 and Figure S6), coagulation (Figure 4, Figures S7 and S8), blood pressure (Figure 4 and Figure S7), and stimulating M1 macrophages (Figure 4, Figures S5, S10 and S11). The pro-inflammatory M1 macrophages phagocytose infected cells and promote apoptosis. Over the course of infection, M1 macrophages are converted to anti-inflammatory M2 macrophages. This process is accompanied by activation of fatty acid oxidation and mitochondrial activity, which are the hallmarks of the M2 macrophage responses (Figure 4, Figures S10 and S11). On the other hand, activation of the complement system is incomplete, potentially leading to a reduced antibody activity (Figure 4 and Figure S5B). Furthermore, filovirus infections were accompanied by downregulation of blood pressure (Figure 4 and Figure S7) and the coagulation system (Figure 4 and Figure S9). The infections are also accompanied by shift in genes regulating iron homeostasis (Figure 4 and Figures S7–S11), which are generally consistent with a greater activation of the M2 macrophage response in case of EBOV infections. The infections resulted in activation of CD8+ T cell response (Figure S5C), presumably contributing to clearance of the infections. The dotted boundaries indicate at pathways which responded to filovirus infections in bats differently from human.

To reveal molecular mechanisms that underlie the resistance of bats to the disease caused by MARV and EBOV, we developed a model of bat response to filovirus infections (Figure 5). Our prior observations suggested that the innate responses to filoviruses are quite similar in human and bat cells [27]. These structural and functional similarities suggest that the ability of bats to tolerate infections with multiple viral pathogens without a disease is unlikely to be explained by features of the interferon response. These data agree with the recently published study on MARV infection in ERBs [78]. Interestingly, the genes postulated to play role contributing to the unique biology of bats by the existence of a distinct immunomodulatory mechanisms to control viral infections highlighted in [25] (NK receptors, MHC class I genes, and certain type I interferons) were not responsive to filovirus infections in our study. Interestingly, the changes in genes expression detected in livers (Figure 4) were generally observed in spleens, although with some exceptions (Figure S2B), and in kidneys, more differences compared to the pattern of expression in liver were observed (Figure S2A).

A key feature of filovirus infection in humans is an inflammatory response leading to the expression of APPs and stimulation of M1 macrophages. In humans, a major APP protein is CRP, which binds to microorganisms, including viruses, assists in complement binding to foreign and damaged cells, and enhances phagocytosis by macrophages (opsonin-mediated phagocytosis) [79]. We found no evidence of CRP expression in bats by mRNA-seq data, while other APPs were conserved and are listed in Table 1. It is possible the lack of CRP response contributes to a lowered inflammation, explaining the resilience of bats to infections by many viruses. As a counterargument, we must keep in mind that in mice, CRP is not an acute phase protein [80]. 

We found evidence in the response of complement genes that an effector component of the antibody response may be weakened by incomplete complement activation. This is consistent with the previous reports that antibody-mediated virus neutralization is not the dominant mechanism of filovirus clearance in ERBs [81]. The robust CD8^+^ T cell activity implied by our mRNA-seq data suggests that control and clearance of filovirus infection in bats may instead depend upon a robust T cell response. This is consistent with what is known in humans, where individuals who recover from filovirus infections tend to mount robust T cell responses [82,83,84], and have higher levels of CD40L expression, a marker for T cell activity [84], although recovery of humans from filovirus infections also correlates with induction of the antibody response [85].

The macrophage response was one of the more notable points of divergence between the human response to filovirus infection and what we observed in infected bats. We identified markers of both M1 and M2 macrophages in ERBs infected with MARV, suggesting that macrophage populations in the animals were in the process of transition towards the anti-inflammatory M2 state, associated with tissue repair and regeneration as opposed to the classic pro-inflammatory M1 state. In particular, the modulation of the innate response facilitated by M2 macrophages is important for T cell mediated clearance of viruses [65]. In EBOV-infected animals, where viral replication was far more limited, our data indicate that the macrophage population was further along in the transition to M2 polarization by the time of euthanasia, likely due to the viral replication being successfully controlled. The generalized anti-inflammatory state observed in bats during filovirus infection, especially the early transition towards M2 macrophage polarization, may suggest a way to prevent the immunopathology associated with filovirus infections in humans, including cytokine storm and DIC. Supporting this, an mRNA-seq study conducted with PBMCs isolated from EBOV-infected humans found that individuals who succumbed to disease showed stronger upregulation of interferon signaling and acute phase response-related genes than survivors during the acute phase of infection [86], suggesting that a moderated innate response improves outcomes in filovirus infection. Furthermore, pharmacological inhibition of toll-like receptor 4 signaling promotes survival in an animal model of filovirus infections [87]. 

Our data suggest that the bat vascular response to infection differs from that in humans. Humans infected with MARV or EBOV frequently present with hemorrhagic manifestations and dysregulated coagulation in the form of DIC [88]. We identified transcriptional patterns consistent with vasodilation and reduced the potential for coagulation, which could result in a state of low blood pressure and reduced coagulation. This state may be protective, as it might be expected to prevent DIC. These findings are consistent with results from a study in humans infected with EBOV which analyzed 55 biomarkers in blood and found that viremia was associated with elevated levels of tissue factor and tissue plasminogen activator, consistent with coagulopathy [89]. 

Our results suggest that reducing the hyperinflammatory response [87] or controlling coagulopathy [90] in humans during filovirus infection may have a therapeutic benefit by preventing damage to the host and allowing other processes to clear the infection. This could be achieved by inhibiting IL-6 signaling either by targeting the cytokine (by Clazakizumab, Olokizumab, Sirukumab, or Siltuximab), or its cognate receptor (by Tocilizumab or Sarilumab), or its trans-signaling by blocking the soluble receptor (sIL-6R) (by Olamkicept) [91]. Strikingly, a recent COVID-19 study demonstrated Tocilizumab reduced the likelihood of progression to the composite outcome of mechanical ventilation or death [92].

We demonstrated that in bats, filovirus infections upregulate MGST1 and MGST2 (Table 1), which, based on its function in humans, likely induce leukotrienes (LTC4) and prostaglandin E, both of which induce inflammation [42]. This is a potential druggable target, as these molecules are targeted by several therapeutic agents. Thus, inflammation caused by filovirus infections could also be potentially targeted by another class of anti-inflammatory agents such as LTC4 inhibitors, used to treat asthma.

Our results also suggest that upon filovirus infections, bats may naturally vasodilate and reduce their blood pressure (mimicking the action of ACE inhibitors), while the endothelial system becomes anti-thrombotic. This suggestion is consistent with the results of the field trials of ACE inhibitors and statins in human Ebola virus disease that have already demonstrated some success [93]. The potential involvement of vasodilation also suggests that prostaglandin I_2_ (PGI_2_, known as the drug Epoprostenol), a powerful vasodilator and anti-coagulant that acts by binding to the prostacyclin receptor [94], could be investigated in human filovirus infections as a means of emulating the physiological conditions (low blood pressure and coagulation) that our data suggest may have protective effects.

In humans, high levels of HAMP cause iron to be sequestered in the liver, reducing levels of iron in blood (lower ferritin). Our observations indicate that in EBOV-infected bats, high HAMP expression is decoupled from the levels of iron, as both ferritin and HAMP are induced. Thus, HAMP inhibitors, which are used to treat anemia, might recreate in humans the state seen in bats under filoviral infection.

The changes in gene expression patterns that we have observed in infected bats suggest that the interconnected pathways regulating coagulation, vasodilation, iron homeostasis, inflammation, the interferon response, and the adaptive response contribute to the unique response of bats to filovirus infection. This response appears to be avoiding immunopathology by tempering of the inflammatory response to infection. In particular, the anti-inflammatory state (macrophages in the M2 state) and the altered state of the blood-related physiological systems seem to be important in preventing pathology and facilitating the ultimate clearance of the viruses. The filobat website [30], developed to accompany this paper, allows easy exploration of our data and comparisons with future studies that might be performed. The unexpected features of bat responses to filovirus infections may aid in the development of new strategies to effectively mitigate and treat the disease caused by filovirus infections in humans.

## Figures and Tables

**Table 1:** Acute phase proteins in livers respond strongly to inflammatory cytokines.

Positive APPs	MARV (Fold Change)	EBOV (Fold Change)	Uninfected (tpm)
Serum Amyloid A 1 (SAA1)	21×	3×	6858
Serum Amyloid A 2 (SAA2)	39×	5×	440
Ceruloplasmin (CP)	10×	5×	129
HAMP *	8.5×	10×	211
Orosomucoid 2 Alpha1-Acid glycoprotein (ORM2 *)	34×	47×	14
Microsomal Glutathione S-Transferase MGST1	4×	4×	277
MGST2 *	11×	16×	5.5
MGST3	0.4×	0.7×	461
Fibrinogen (FGA)	2×	1×	1277
Fibrinogen (FGB)	2×	1×	9007
Fibrinogen (FGG)	1×	1×	6070
C4B	2×	1×	1015
C3P1	6×	1×	31
Haptoglobin (HP)	1.1×	0.7×	15,906
Alpha2-Macroglobulin (A2M)	1.3×	1×	409
C-reactive protein (CRP)	N/A	N/A	N/A
**Negative APPs**			
Albumin (ALB)	0.6×	1.2×	51,400
Transferrin (TF)	1×	1×	22,856
Transthyretin (TTR)	2×	2×	1
Retinol Binding protein (RBP4)	0.5×	0.6×	3107

Basal expression levels in tpm units are shown in the Uninfected column. The fold change upon infection is shown in the MARV and EBOV columns. SAA1/2 and CP were highly expressed in livers normally and are also upregulated by filovirus infections, with a greater upregulation by MARV. ORM2 and MGST2 are upregulated, but from a low basal expression level. TF was highly expressed in all samples, but the expression did not change in response to filoviral infections, while TTR was not expressed in any of the samples. A similar inflammation was detected in the liver upon both MARV and EBOV infection, despite the lack of viral transcripts in the liver of EBOV-infected animals. CRP, used as a marker for acute phase response in humans, is highlighted in gray to emphasize that it does not appear to be expressed in this bat species and might be absent in bats altogether. * Bat genes divergent from their respective human homologs.

## Data Availability

The raw sequencing reads are deposited in GEO, accession number GSE152728. Csv files of all data underlying the balloon plots, a fasta-format file containing all the mRNA sequences used in the analysis and tools for analysis and exploration of data are deposited in the filobat website [31].

## Supplementary Materials

The following supporting information can be downloaded at: https://www.mdpi.com/article/10.3390/v15020350/s1, Figure S1:A multidimensional scaling (MDS) plot of the merged gene expression data from kidneys of MARV-infected, EBOV-infected and uninfected bats.; Figure S2: A. Differential expression of genes belonging to the indicated pathways by MARV and EBOV infections of bats in kidneys; B: Differential expression of genes belonging to the indicated pathways by MARV and EBOV infections of bats in spleens; Figure S3: Pathway analysis of innate response to filovirus infections in bats; Figure S4: Induction of interferon stimulated genes (ISG) in livers of filovirus-infected bats; Figure S5: Tissue regeneration, the complement system and CD8+ T cells; Figure S6: Iron metabolism; Figure S7: Blood pressure pathways; Figure S8: The coagulation pathway; Figure S9: Details of the coagulation pathway; Figure S10: Pathway analysis related to macrophage polarization during filovirus infections; Figure S11: Macrophage activation; Table S1: List of bat samples profiled using mRNA-seq; Table S2: MARV transcripts detected using mRNAseq in tissues of MARV-infected bats; Table S3: Divergent pathways and genes upregulated by MARV and EBOV infection:.vascular, mitochondrial, oxida-tion-reduction, innate immunity, T cell activity, complement, digestion, toxins, inflammation and tissue regenera-tion.; Table S4: Divergent pathways and genes downregulated by MARV and EBOV infection:.mitochondrial activity, vascular function, inflammation, innate immunity, lipids, toxins, macrophage activation, splicing, T cell activity, metabolism, digestion, macrophages and apoptosis; Table S5: Divergent pathways and genes upregulated by MARV but not EBOV infection: macrophages, complement, apoptosis, mitochondrial respiration, innate immunity, inflammation, digestion, T cells and the vascular system; Table S6: Divergent pathways and genes downregulated by MARV but not EBOV infection: mitochondrial, vascular, inflammation, digestion, innate immunity, complement, apoptosis and splicing; Table S7: Divergent pathways and genes upregulated by EBOV but not MARV infection: vascular function, inflammation, mitochondria, lipid metabolism, tissue regeneration; Table S8: Divergent pathways and genes downregulated by EBOV but not MARV infection: innate immunity, coagulation and digestion; Table S9: Correlations between various samples based on expression levels of genes.
