# Supplementary material for: Marburg and Ebola Virus Infections Elicit a Complex, Muted Inflammatory State in Bats
Source: Viruses. 2023 Jan 26;15(2):350. doi: 10.3390/v15020350 (PMC9958679; doi:10.3390/v15020350)
Supplement: Supplementary file 1 [file viruses-15-00350-s001.zip › viruses-2120751-supplementary.pdf]

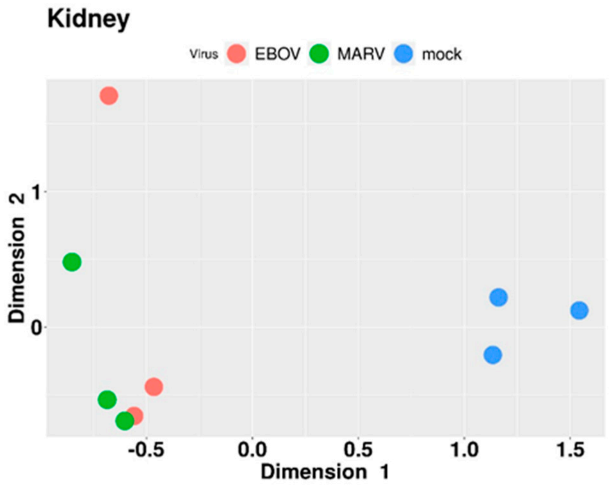

Figure S1. A multidimensional scaling (MDS) plot of the merged gene expression data from kidneys of MARV-infected, EBOV-infected and uninfected bats. The plot shows a clear separation between MARV infections, EBOV infections and the uninfected samples. The virus-specific signatures in this plot, along with that for livers and spleens (Fig. 2), demonstrate that the identified transcriptional responses to filovirus infections extends to the whole animal.

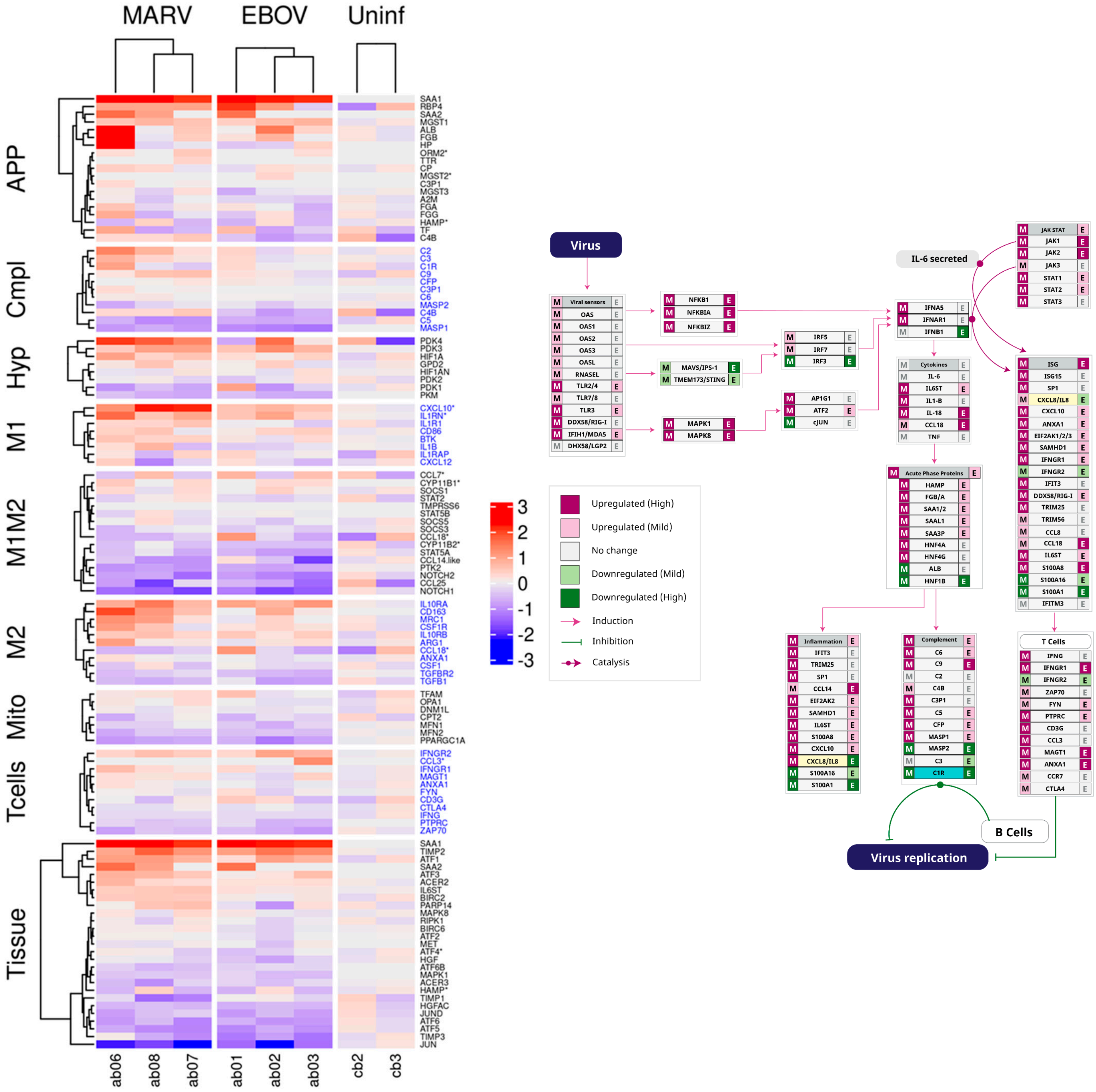

MARV
EBOV
Uninf
APP
Cmpl
Hyp
M1
M1M2
M2
Mito
Tcells
Tissue
SAA1 RBP4 SAA2 MGST1 ALB FGB HP ORM2* TTR CP MGST2* C3P1 MGST3 A2M FGA FGG HAMP* TF C4B
C2 C3 C1R C9 CFP C3P1 C6 MASP2 C4B C5 MASP1
PDK4 PDK3 HIF1A GPD2 HIF1AN PDK2 PDK1 PKM
CXCL10* IL1RN* IL1R1 CD86 BTK IL1B IL1RAP CXCL12
CCL7* CYP11B1* SOCS1 STAT2 TMPRSS6 STAT5B SOCS5 SOCS3 CCL18* CYP11B2* STAT5A CCL14.like PTK2 NOTCH2 CCL25 NOTCH1
IL10RA CD163 MRC1 CSF1R IL10RB ARG1 CCL18* ANXA1 CSF1 TGFBR2 TGFB1
TFAM OPA1 DNM1L CPT2 MFN1 MFN2 PPARGC1A
IFNGR2 CCL3* IFNGR1 MAGT1 ANXA1 FYN CD3G CTLA4 IFNG PTPRC ZAP70
SAA1 TIMP2 ATF1 SAA2 ATF3 ACER2 IL6ST BIRC2 PARP14 MAPK8 RIPK1 BIRC6 ATF2 MET ATF4* HGF ATF6B MAPK1 ACER3 HAMP* TIMP1 HGFAC JUND ATF6 ATF5 TIMP3 JUN
ab06 ab08 ab07 ab01 ab02 ab03 cb2 cb3
3 2 1 0 -1 -2 -3
Virus
Viral sensors
OAS OAS1 OAS2 OAS3 OASL RNASEL TLR2/4 TLR7/8 TLR3 DDX58/RIG-I IFIH1/MDA5 DHX58/LGP2
NFKB1 NFKBIA NFKBIZ
MAVS/IPS-1 TMEM173/STING
MAPK1 MAPK8
IRF5 IRF7 IRF3
AP1G1 ATF2 cJUN
IL-6 secreted
IFNA5 IFNAR1 IFNB1
Cytokines
IL-6 IL6ST IL1-B IL-18 CCL18 TNF
JAK STAT
JAK1 JAK2 JAK3 STAT1 STAT2 STAT3
ISG
ISG15 SP1 CXCL8/IL8 CXCL10 ANXA1 EIF2AK1/2/3 SAMHD1 IFNGR1 IFNGR2 IFIT3 DDX58/RIG-I TRIM25 TRIM56 CCL8 CCL18 IL6ST S100A8 S100A16 S100A1 IFITM3
Acute Phase Proteins
HAMP FGB/A SAA1/2 SAAL1 SAA3P HNF4A HNF4G ALB HNF1B
Upregulated (High)
Upregulated (Mild)
No change
Downregulated (Mild)
Downregulated (High)
Induction
Inhibition
Catalysis
Inflammation
IFIT3 TRIM25 SP1 CCL14 EIF2AK2 SAMHD1 IL6ST S100A8 CXCL10 CXCL8/IL8 S100A16 S100A1
Complement
C6 C9 C2 C4B C3P1 C5 CFP MASP1 MASP2 C3 C1R
T Cells
IFNG IFNGR1 IFNGR2 ZAP70 FYN PTPRC CD3G CCL3 MAGT1 ANXA1 CCR7 CTLA4
B Cells
Virus replication
M
E

A

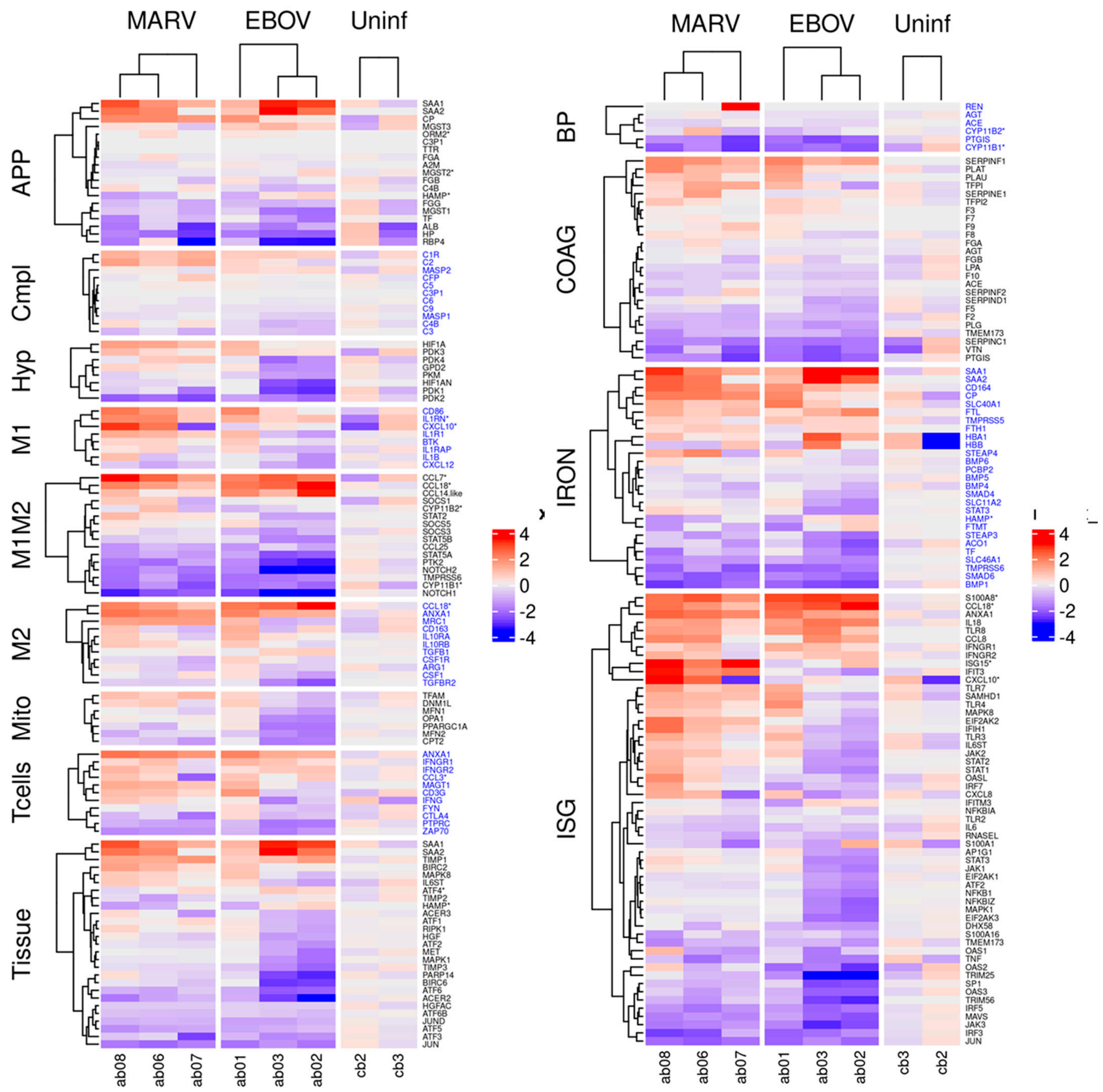

B

Figure S2. (A). Differential expression of genes belonging to the indicated pathways by MARV and EBOV infections of bats in kidneys. Left panel: genes related to acute phase response (APP), complement system (Cmpl), hypoxia (Hyp), tissue regeneration and apoptosis (Tissue), and genes specific to macrophages in the M1 state (M1), M2 state (M2) and, common to M1 and M2 states (M1M2). Right panel: genes related blood pressure (BP), coagulation (COAG), iron homeostasis (IRON) and interferon stimulated genes (ISG). The columns show samples from three MARV-infected bats, three EBOV-infected bats and two uninfected bats. The values are $\log_2$ of the fpm values, with the mean value of the samples from uninfected bats subtracted. The data show virus-specific responses which are not as pronounced as in the liver (Fig. 4); the responses are greater for MARV. Renin, in the BP group of gene, is only expressed in kidneys. There is a strong response in the SAA1/2 APP genes. * Genes whose bat versions are diverged from their human counterparts. Alternate blocks of gene names are colored black/blue to enable easy visual

distinction of the blocks. **(B). Differential expression of genes belonging to the indicated pathways by MARV and EBOV infections of bats in spleens.** Left panel: genes related to acute phase response (APP), complement (Cmpl) , hypoxia (Hyp), tissue regeneration / apoptosis (Tissue), and genes specific to macrophages in the M1 state (M1), M2 state (M2) and, common to M1 and M2 states (M1M2). Right panel: genes for blood pressure (BP), coagulation (COAG), iron homeostasis (IRON) and Interferon stimulated genes (ISG). The columns show the samples from three MARV-infected bats, three EBOV-infected bats and two uninfected bats. The values are log2 of the fpm values, with the mean value of the uninfected samples subtracted. The virus-specific response was not as pronounced as in the liver (Fig. 4) or kidneys (Fig. S2A), with larger effects in the case of MARV. A strong response in expression of the SAA1/2 APP genes was observed. Sting (TMEM173) expression was only detected in the spleen. * Genes whose bat versions are diverged from their human counterparts. Alternate blocks of gene names are colored black/blue to enable easy visual distinction of the blocks.

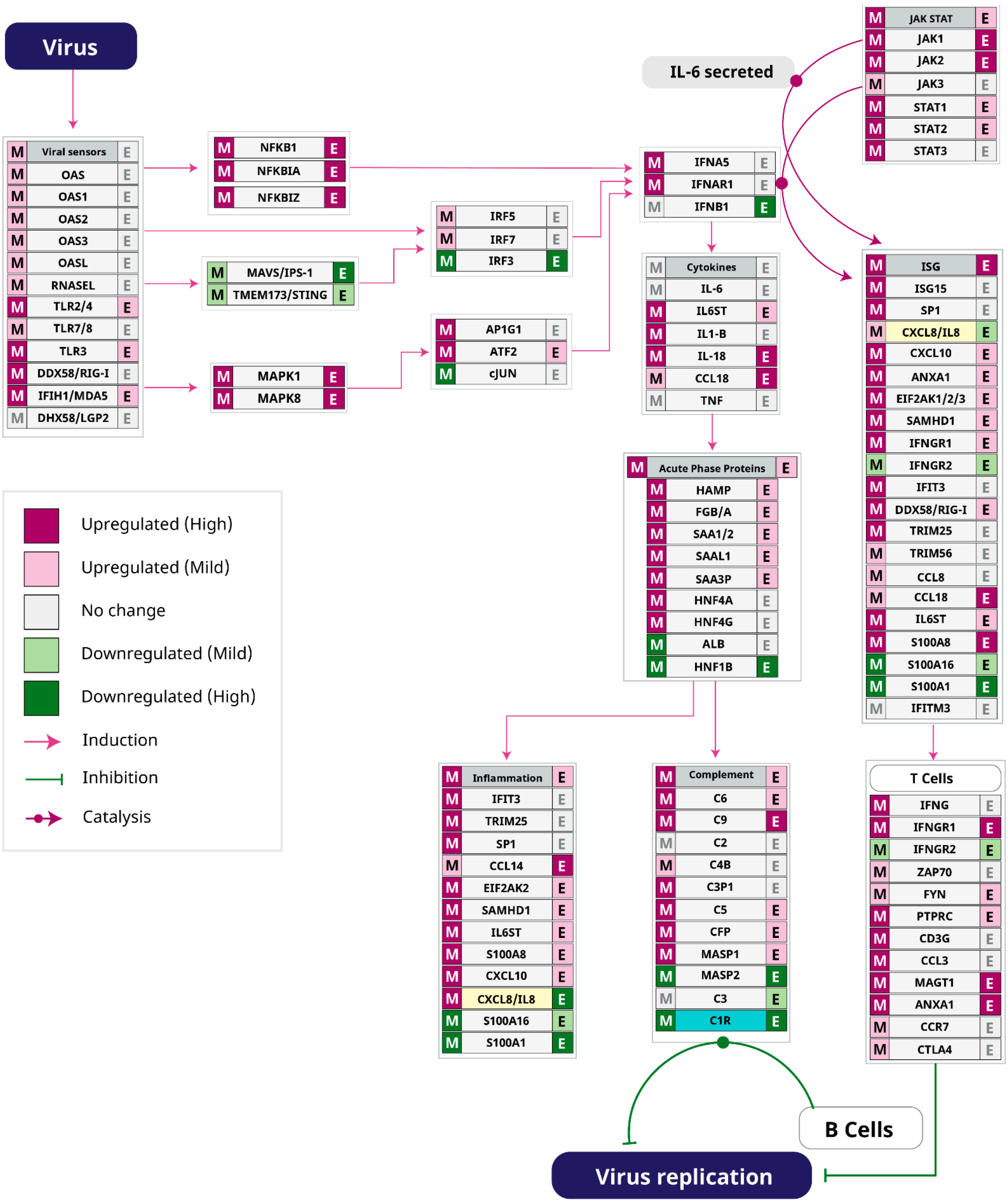

Figure S3. Pathway analysis of innate response to filovirus infections in bats. Viral RNA and proteins are detected by innate immune sensors, leading to upregulation of type I interferons (IFNα and IFNβ) and interferon stimulated genes (ISG) through the JAK-STAT pathway. Cytokines and chemokines create an anti-viral state in the cell. Inflammation (in part mediated by IL-6) triggers expression of acute phase proteins (APPs). Secretion of interferon-**γ** gamma enables an adaptive immune response. In human and bat cells interferon responses to the filoviruses were mostly similar, with a few virus-specific differences. VP35 from both viruses interferes with IRF3/7, while EBOV VP24 inhibits STAT1, and MARV-VP40 inhibits JAK1, which can lead to differences in responses to MARV and EBOV. The robust innate response to filovirus infection in bats and humans suggests any constitutive expression of innate response genes in bats is unlikely to be relevant to explain the difference in the pathogenesis. **Fig. 4** shows the relative changes in expression upon infection for these genes. Here, colored bands flanking gene names depict the effect of filovirus infection on gene expression for MARV (left) and EBOV (right).

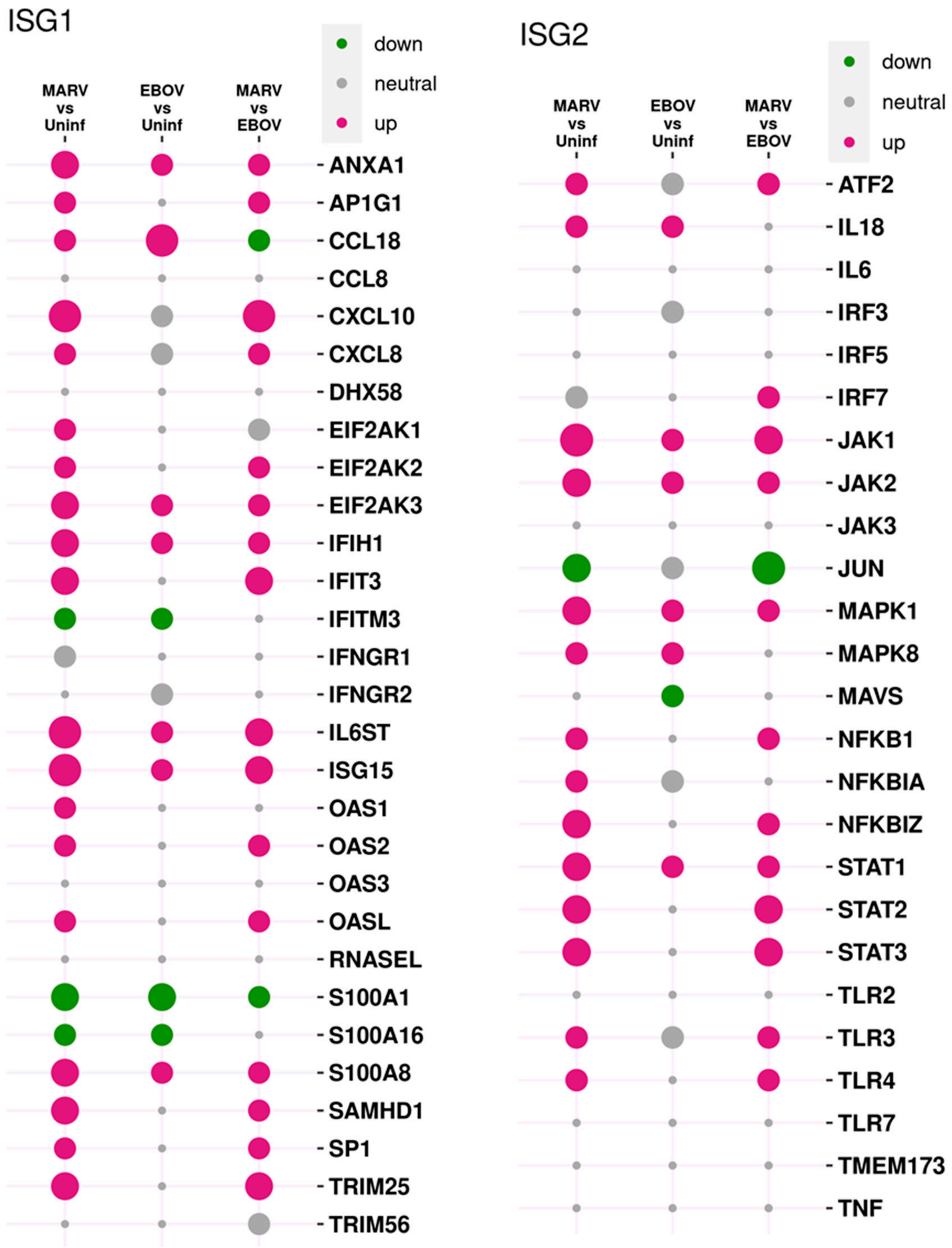

Figure S4. Induction of interferon stimulated genes (ISG) in livers of filovirus-infected bats. MARV and EBOV elicit a strong innate response driven by IFNs. The balloon plot shows comparisons of responses of ISG genes to MARV and EBOV against uninfected animals and against each other. The radius of circle is proportional to $\log_2$(ratio), red is $\log_2(\text{ratio}) > 0.6$, green is $\log_2(\text{ratio}) < -0.6$, gray is $-0.6 < \log_2(\text{ratio}) < 0.6$. The response is stronger in the MARV infected animals, corresponding to the greater replication of MARV as compared to EBOV in ERBs.

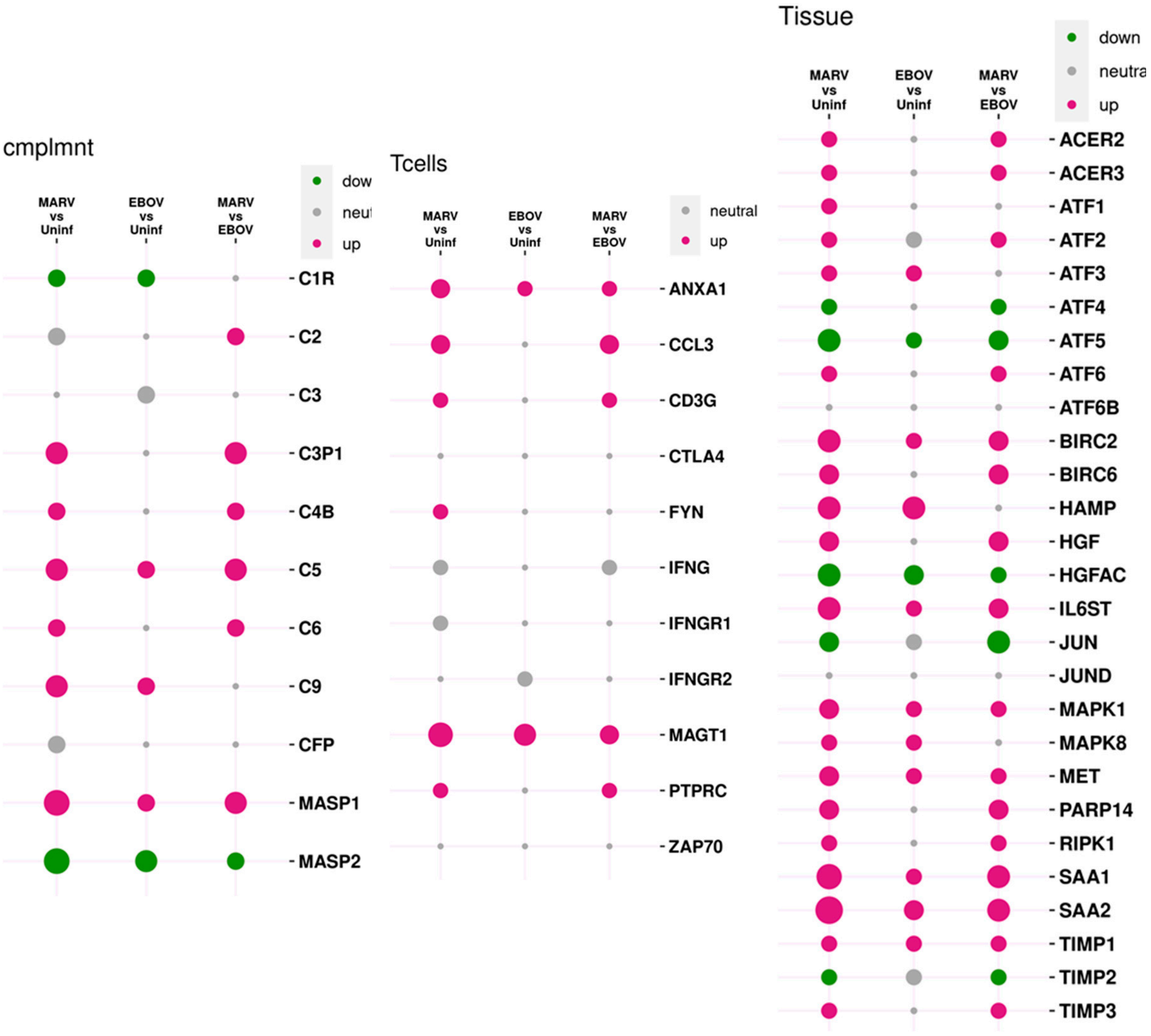

A B C

Figure S5. Tissue regeneration, the complement system and CD8$^+$ T cells. Responses of genes in MARV and EBOV infected bats against uninfected bats and against each other. Balloon plots with the radius of circles proportional to $\log_2$ (ratio), gray is used when absolute values of $\log_2$ (ratio) < 0.6. **A.** The complement system: filovirus infections downregulated C1R, C3 and MASP2, likely leading to reduced antibody activities. **B.** CD8$^+$ T cell genes: most of them were upregulated by filovirus infections, indicating at CD8$^+$ T cell response, which is likely to be involved in clearance of filovirus infections in bats **C.** Tissue regeneration: filovirus infections in bats eventually trigger tissue regeneration, reflected in the activation of M2 macrophages, which are anti-inflammatory and promote regeneration of tissues. The greater effect of MARV infection as compared to EBOV is consistent with the higher viral loads.

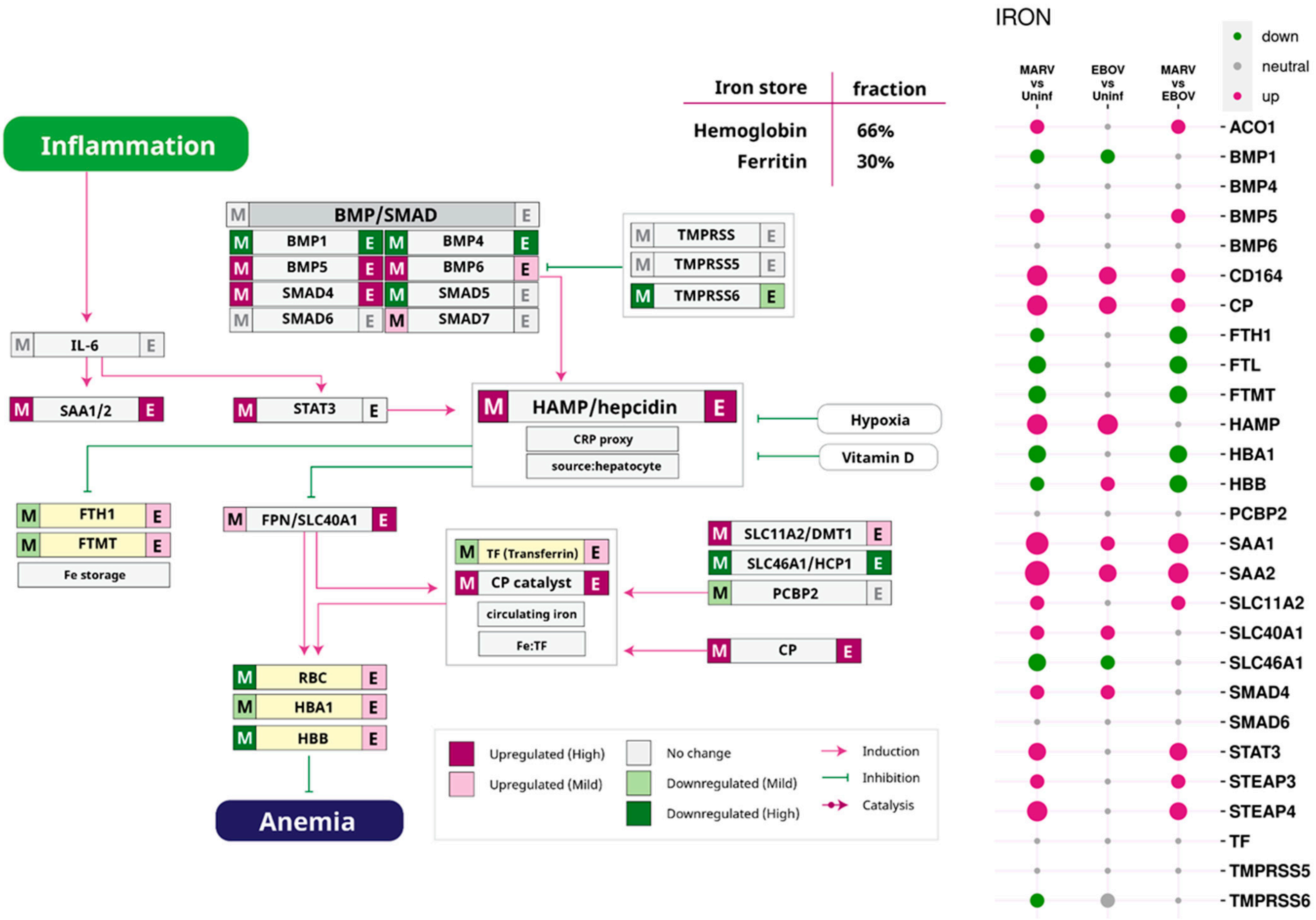

A B

Figure S6. Iron metabolism. A. Pathway analysis of genes involved in iron homeostasis. Colored bands flanking gene names depict the effect of filovirus infection on gene expression for MARV (left) and EBOV (right), with upregulation depicted by shades of red and downregulation depicted by shades of green. The lines show induction (red) and inhibition (green). Filovirus infections lead to upregulation of HAMP. In MARV infection, this may lead to a decrease in blood iron levels and impaired hematopoiesis, but in EBOV infection, this regulation seems to be broken, leading to a high iron state. These differences between MARV and EBOV infections are consistent with the greater replication of MARV in ERBs. ACO1 is upregulated during filovirus infections, suggesting the cytosol has abundant iron. **B.** The balloon plot compares responses of genes in MARV and EBOV infected bats against uninfected bats and against each other. The radius of circle is proportional to $\log_2$(ratio), gray is used when absolute values of $\log_2$(ratio) < 0.6.

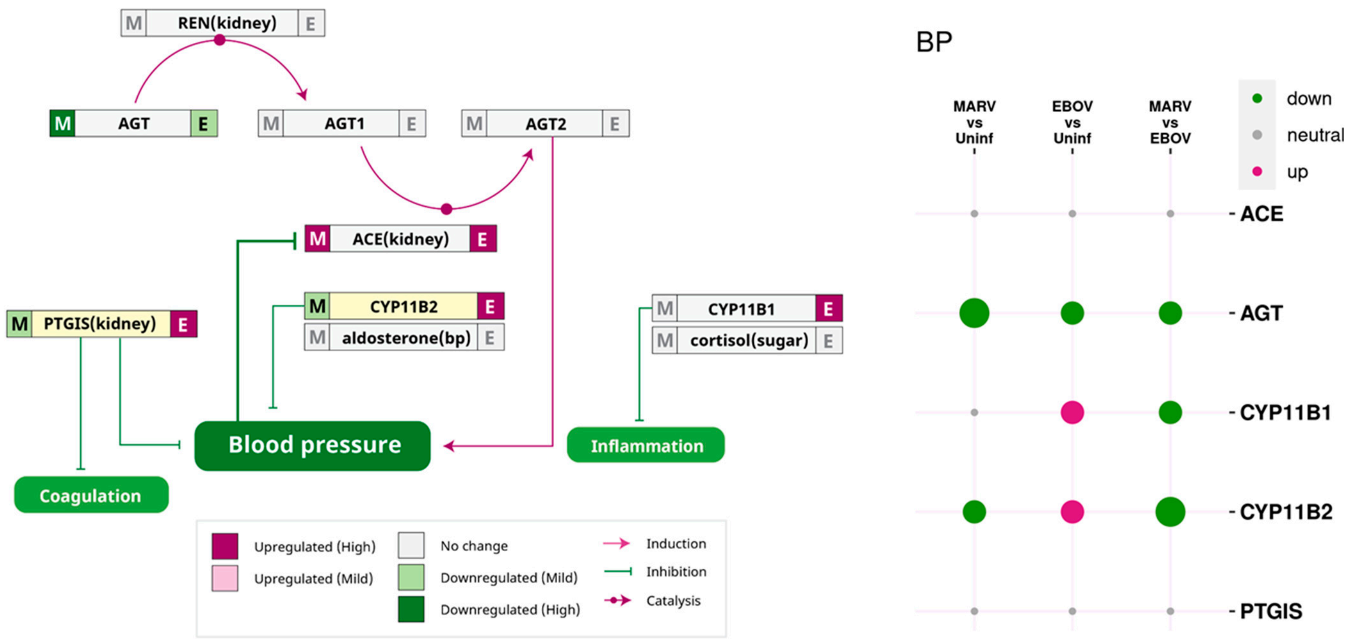

A B

Figure S7. Blood pressure pathways. A. Analysis of pathways associated with renin (REN) and angiotensin I-converting enzyme (ACE) which catalyze conversion of AGT to AGT2, which in turn constricts blood vessels to increase pressure. Filovirus infections in ARBs lowered expression of AGT but increased PTGIS, which reduces blood pressure and coagulation and increased ACE, which is a sensor of low blood pressure. Thus, filovirus-infected bats induces an anti-coagulative state with low blood pressure. The colored bands on either side of the gene names depict the effects of MARV (left) and EBOV (right) infections on expression of the indicated genes. **B.** The balloon plot compares responses of genes to MARV and EBOV compared to uninfected animals and against each other. The radius of circle is proportional to $\log_2$ (ratio), gray is used when absolute values of $\log_2$ (ratio) < 0.6.

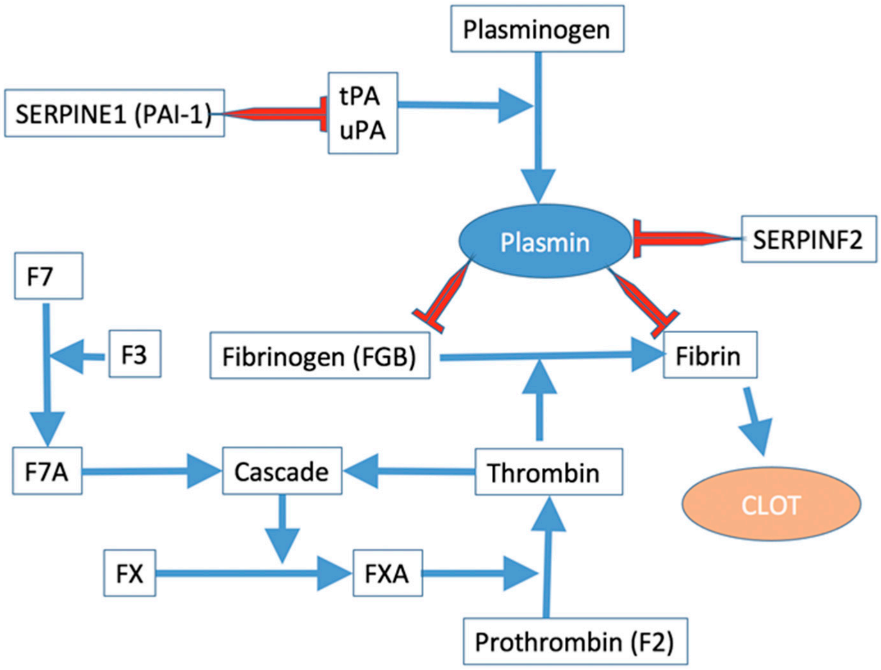

Figure S8. The coagulation pathway. The coagulation pathway involves a cascade of activations that eventually results in fibrin formation that is part of

the clot. There is an opposing process where plasmin degrades fibrinogen and fibrin thereby dissolving clots. The figure only shows processes that are relevant to the discussion in the paper. The red lines show inhibition of a process or degradation, while the blue arrows signify enhancement of the process/product. The process starts with tissue factor (F3) activating F7 (to form F7A) which eventually leads to the formation of Thrombin, which facilitates fibrin synthesis**. Fig.** **S9** shows the expression changes in coagulation pathway genes upon filovirus infections.

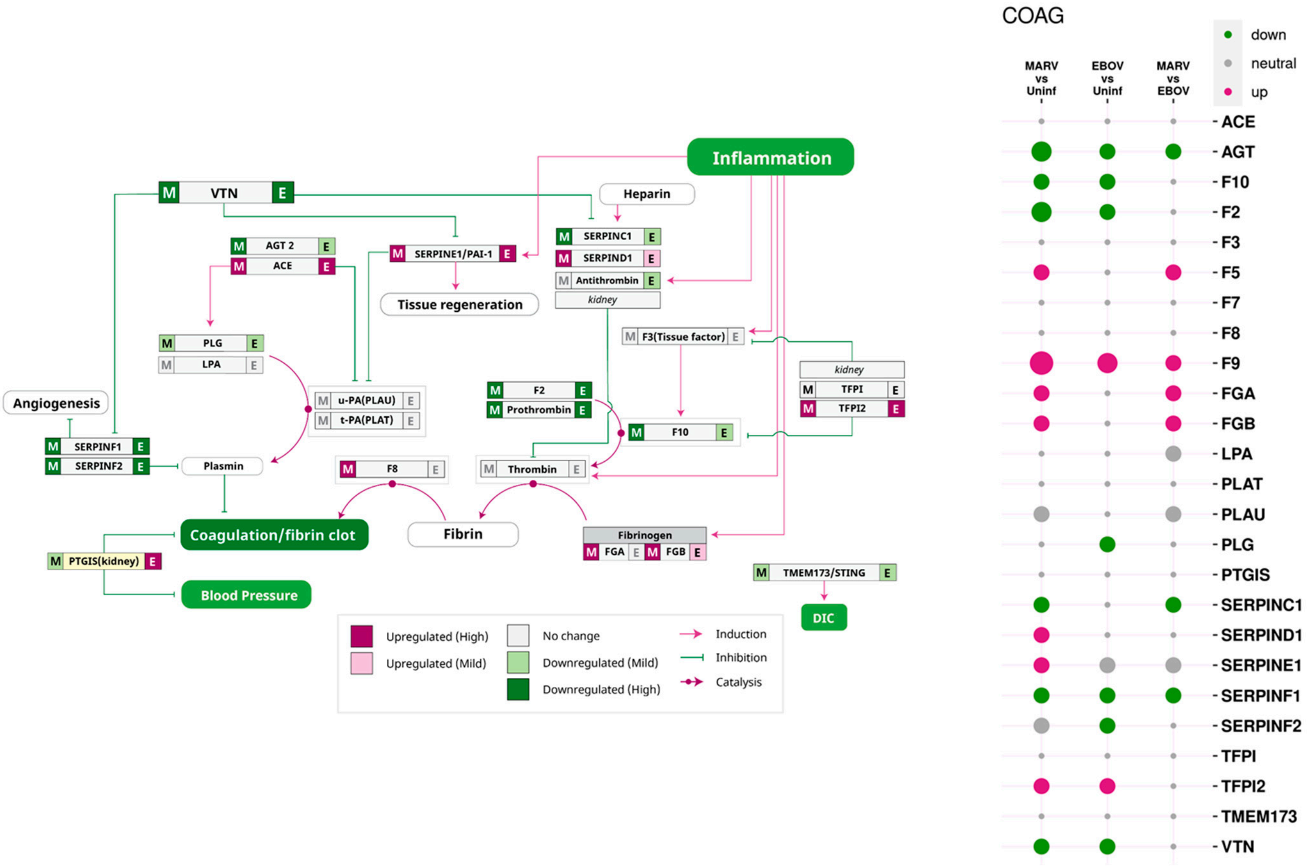

A B

Figure S9. Details of the coagulation pathway. A. Analysis of pathways associated with coagulation cascade (shown in **Fig. S8**). Except for F3, the tissue factor, all other proteases in the cascade require activation. The serine protease plasmin opposes this process by attacking the fibrin mesh to dissolve clots. Urokinase-type plasminogen activator (uPA, PLAU gene) activates plasminogen to generate plasmin. SERPINE1 (PAI-1) inhibits the activity of uPA, blocking the creation of plasmin, thereby stabilizing clots. SERPINF2 also stabilizes clots by directly inhibiting plasmin. PTGIS creates prostacyclin, which prevents platelet aggregation, yet another path for inhibiting clot formation and coagulation. Prostacyclin is also a potent vasodilator. Failure in the control of plasmin (e.g., inactivation of SERPINF2 which inhibits plasmin) can lead to hemorrhagic diathesis, while blocking plasmin activity can lead to excessive clotting. Filovirus infections in bats lead to low F2 (which lowers fibrin) and raise levels of PTGIS, which suggest that filovirus-infected bats are in a low coagulation state. **B.** The balloon plot compares responses of genes to MARV and EBOV compared to uninfected animals and against each other. The colored bands on either side of the gene names depict the effects of filovirus infections on gene expression for MARV (left) and EBOV (right) compared to uninfected animals. The radius of circle is proportional to $\log_2$(ratio), gray is used when absolute values of $\log_2(\text{ratio}) < 0.6$.

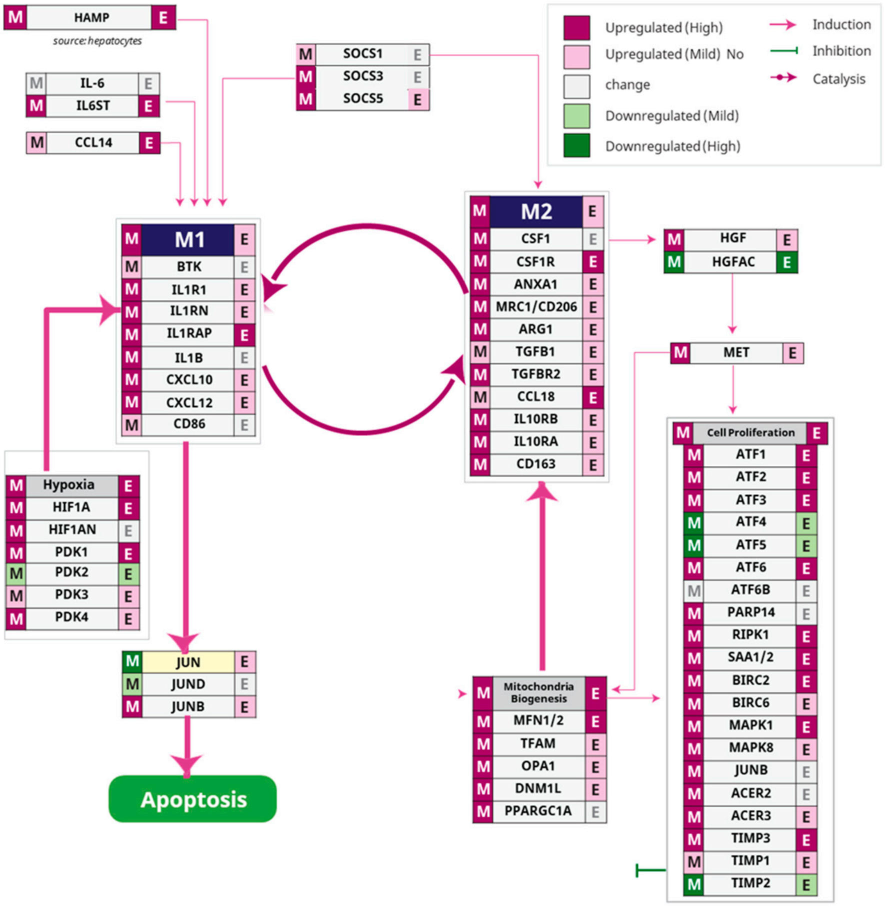

Figure S10. Pathway analysis related to macrophage polarization during filovirus infections. Differential expression of genes involved in macrophage polarization between the pro-inflammatory M1 state and the anti-inflammatory M2 state. Colored bands flanking gene names depict the effects of MARV (left) and EBOV (right), with upregulation depicted by shades of red and downregulation depicted by shades of green. The lines show induction (red) and inhibition (green). Filoviral infections initially lead to proinflammatory state of macrophages (M1) which then transitions to anti-inflammatory state (M2). The differential expression levels of these genes as balloon plots is shown in Fig. S5. HAMP induces macrophages in the proinflammatory M1 state which phagocytose infected cells and induces apoptosis. In contrast, the M2 macrophages induce cell proliferation and tissue regeneration. Different SOCS family molecules serve as molecular switches that control M1/M2 macrophage switch. Fatty acid oxidation and increased mitochondrial numbers and activity indicates an M1 to M2 switch. During filoviral infections, both M1 and M2 states are seen, based on the markers specific to them, but the ratio of M1 to M2 was skewed more towards M2 in EBOV-infected bats case, consistent with the more limited virus replication, while the M1 to M2 transition was still underway in the MARV-infected bats. The switch to the M2 state is probably the key to the resilience of bats during filovirus infections, allowing bats to tolerate filovirus infections without significant adverse effects. There is also a connection between iron metabolism and macrophage M1/M2 polarization with increasing iron (Fig. S6) favoring the M2 state.

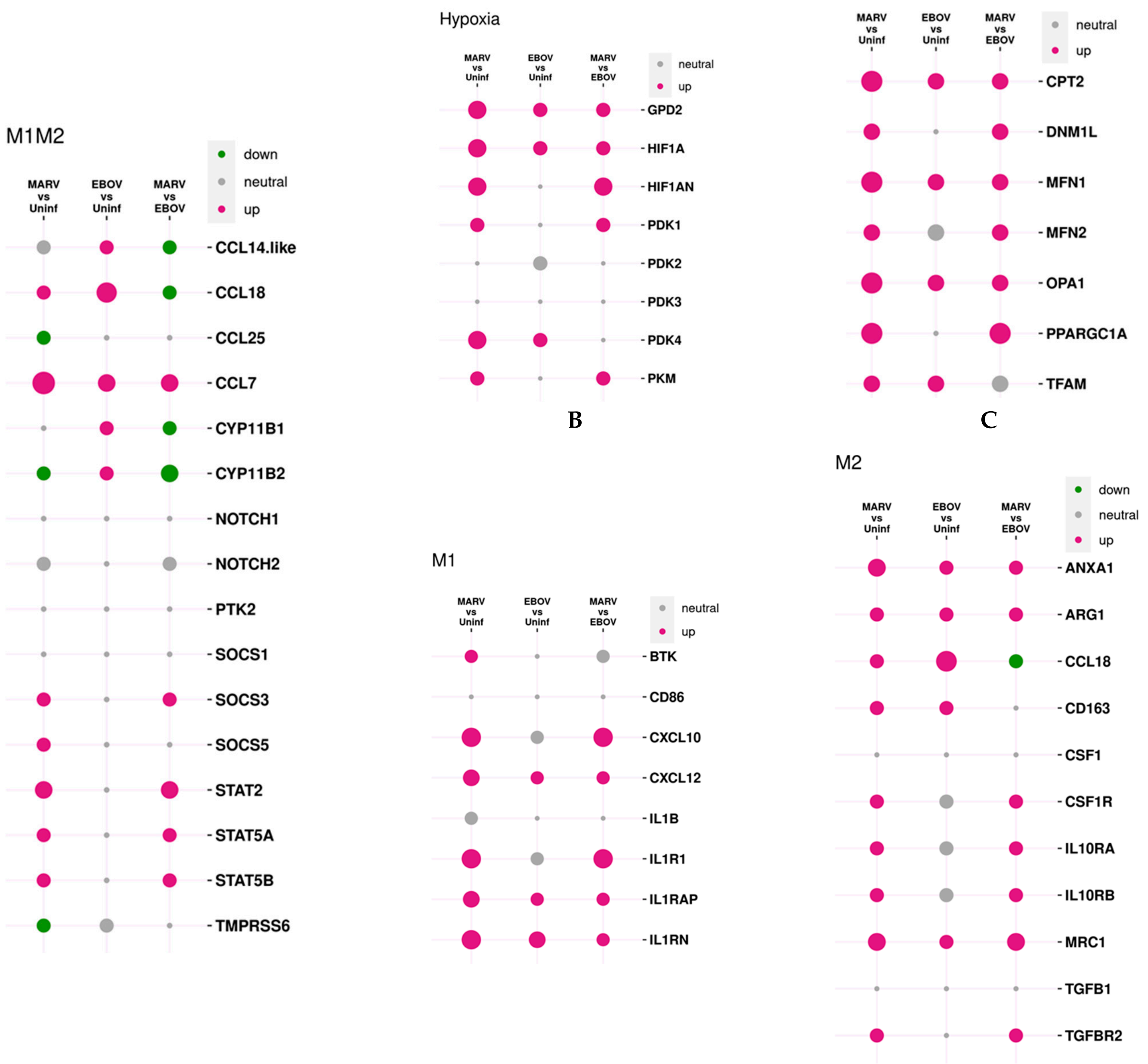

A D E

Figure S11. Macrophage activation. Left panel(**A**): genes common to both M1 and M2 states. Top right panels (**B,C**): genes involved in hypoxia and mitochondrial respiration/abundance. Bottom right panels(**D,E**): genes specific to the M1 and M2 macrophages which show that both M1 and M2 states are activated by filovirus infections with the M1/M2 ratio being higher in MARV infection. The balloon plots shows comparisons of gene expression responses to MARV and EBOV in comparison with uninfected animals. The radius of circle is proportional to $\log_2$ (ratio), gray is used when absolute values of $\log_2$ (ratio) < 0.6. There are more activated macrophages in the MARV-infected bats compared to EBOV-infected bats, consistent with greater level of MARV replication.

Table S1. List of bat samples profiled using mRNA-seq.

Tissue	Uninfected	MARV	EBOV
Liver	3	3	3
Lung	0	3	3
Spleen	3	3	3
Kidney	3	3	3

Large intestine	0	3	3

Table S2. MARV transcripts detected using mRNAseq in tissues of MARV-infected bats.

Organ	ab07	ab08	ab06
Liver	79	8.7	3.1
Spleen	56	8.5	3.4
Large intestine	2.3	10.1	NA
Lung	2.3	0	NA

NA, not analyzed, stands for samples that failed mRNAseq. The values are expressed in tpm. EBOV transcripts are seen only in liver in very low numbers.

Table S3. Divergent pathways and genes upregulated by MARV and EBOV infection:.vascular, mitochondrial, oxidation-reduction, innate immunity, T cell activity, complement, digestion, toxins, inflammation and tissue regeneration.

Gene	Process
	Vascular
HAMP/Hepcidin	Cellular iron ion homeostasis inflammatory induces M1 macrophages
SRGN	Platelet degranulation
AK3	Platelet production nucleoside diphosphate phosphorylation mitochondrial
GLRX	Antioxidant defense system VEGF expression vascular growth
CEBPZOS	Blood cell maturation
	Mitochondrial
MRPL50	Organelle organization
GSTZ1	Detox reduces oxidative stress
	Redox
PECR	Oxidation-reduction process lipid synthesis regeneration cell growth
GALM	Glucose metabolic process
	Macrophages
APOL6	Monocyte to macrophage differentiation lipid metabolism
TIMD4	Expressed by macrophage maintains killer T cell activity
CCL18	Attracts T cells to macrophages cellular response to IFNG
CXCL10	secreted by macrophage in response to IFNG role in hypertension Immune response
CCL3	Macrophage inflammatory protein cellular response to IFNG
PRXL2A	Inhibits production of inflammatory cytokines by macrophages
S100A12	paralog of S100A8 IL-10 induced monocytes macrophages Proinflammatory mast cell chemoattractant innate immune response
PLAC8	Expressed by macrophage phospholipid metabolic process
ISG15	Regulation of IFNG production Mito function in macrophages
CCL7	Regulates macrophages attracts monocytes eosinophils but not neutrophils cellular response to interferon-gamma
	Innate immunity
CLEC4F	Endocytosis pathogen detector

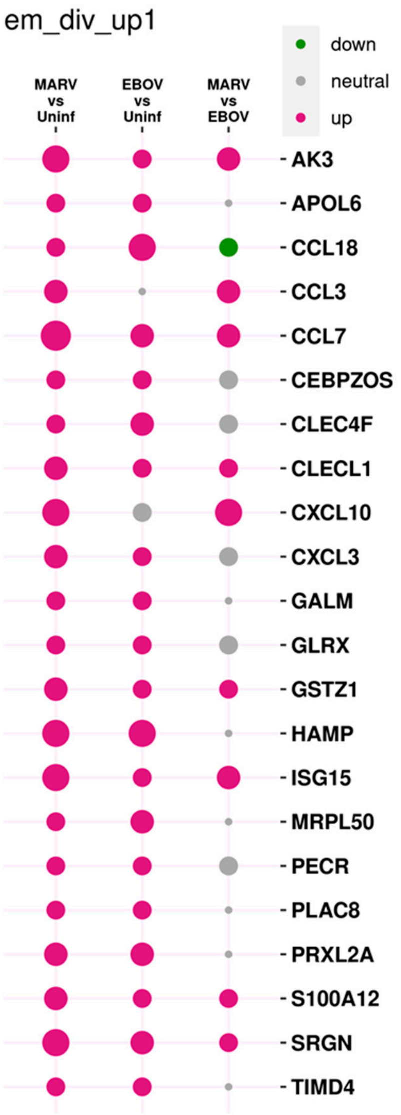

Gene	Process
CLECL1	Expressed by dendritic and B cells enhances IL-4 production regulates immune response
CXCL3	Chemoattractant for neutrophils immune response
**Gene**	**Process**
	T cell
LY6E	T cell development negative regulator of monocytes dampens response
	Complement
MBL2	Activates complement mannose-binding innate immune response
	Digestion
PRB1	Digestion salivary proline-rich protein
	Toxins
UGT2A3	Excretion of toxic compounds flavonoid biosynthetic process
	Inflammation
ORM2	Acute phase reactant regulation of immune system process
IL1RN	IL-1R antagonist inhibits IL1A/B, modulates inflammation immune response
MGST2	Generates LTC4 mediates stress
S100A8	Leukocyte migration involved in inflammatory response
	Tissue regeneration
ACOT13	Essential for cell proliferation mitochondrial function (macrophage M2)
S100P	Cell proliferation, response to organic substance
NUDT16	Positive regulation of cell proliferation
EIF4EBP3	Negative regulation of translational initiation
	Other
ZG16B	Atherosclerosis retina homeostasis
SUB1	Regulation of transcription from RNA polymerase II promoter
UNDEF108	
UNDEF771	

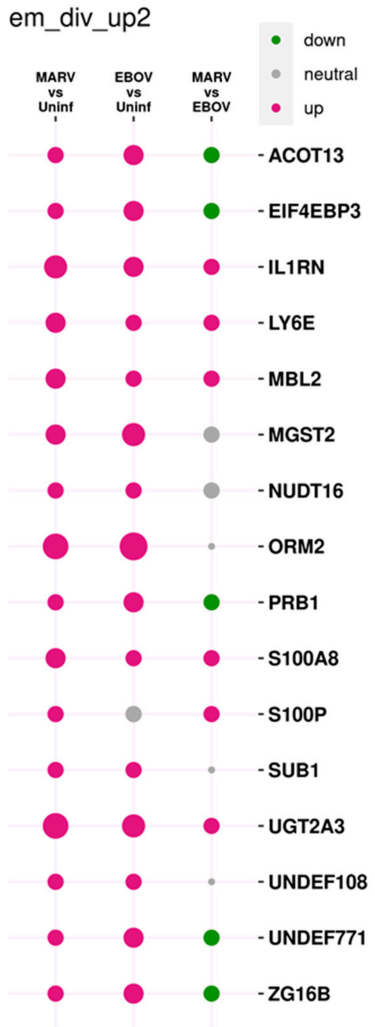

The balloon plot on the right compares responses of genes to MARV-, EBOV-infected and uninfected samples against each other. The radius of circle is proportional to $\log_2$(ratio), red is for positive, green is for negative values and gray is used when absolute values of $\log_2$(ratio) < 0.6.

Table S4. Divergent pathways and genes downregulated by MARV and EBOV infection:.mitochondrial activity, vascular function, inflammation, innate immunity, lipids, toxins, macrophage activation, splicing, T cell activity, metabolism, digestion, macrophages and apoptosis.

Gene	Process
	Mitochondria/oxidation/fatty-acid
MRPL54	Organelle organization
CA3	Nitrogen metabolism bicarbonate transport
RNASEH1	mtDNA replication mutations lead to autoimmunity(T1D)
CHCHD7	Protein import
CHCHD10	Negative regulation of ATP citrate synthase activity
	Vascular
ART4	Blood group antigens protein ADP-ribosylation
HRG	Platelet degranulation Histidine-rich glycoprotein
MEG3	Negative regulation of VEGF receptor signaling pathway
TMEM80	Increased transferrin (TF) endocytosis
	Inflammation
AHSG	Regulation of inflammatory response
CRELD2	ER-stress response
TEX264	Stress , elevated platelet cytosolic Ca2+ responsive
	Innate immunity
BTBD6	Class I MHC-mediated antigen processing/presentation
C8G	Complement Complement component C8 gamma chain
EEF1D	Positive regulation of I-kB kinase/NF-kB signaling
MIIP	Down-regulates NFKB2 and ICAM1 inhibition of migration/invasion
TMEM80	Increased vaccinia virus (VACV) infection Decreased NF-kB reporter expression
CRIP1	Cysteine-rich protein 1
	Lipids
MOGAT1	Triacylglycerol biosynthesis and Metabolism
	Toxin
GSTA5	Detoxification glutathione metabolic process
SLC17A2	Sodium/anion cotransporter family ossification
	Macrophages
RNASET2	Chemoattractants for macrophages and modulate the inflammatory processes
	Vascular (iron)
ATP6V0D2	Cellular iron ion homeostasis
BSG	Carries OK antigens on red blood cells:cell surface receptor Signaling pathway:inflammation
LCN2	Sequesters iron (antibacterial) iron/toxin transport cisplatin resistance innate immune response
**Gene**	**Process**
	Splicing
SNRPN	mRNA splicing via spliceosome
	T cell
GPX4	Role in primary T-cell response to viral infection protects T-cells from ferroptosis supports T-cell expansion mitochondrial

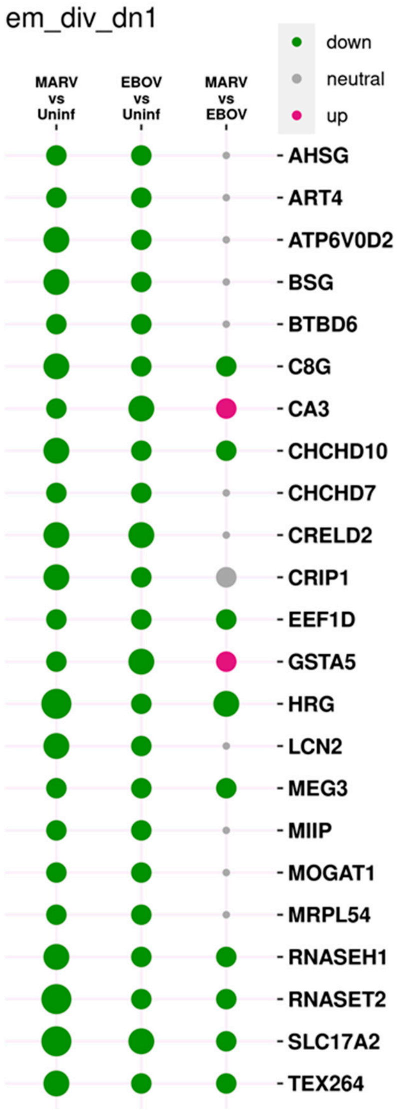

	Metabolism
TSTD1	Thiosulfate sulfur transferase/rhodanese-like
NUDT14	Hydrolyzes UDP-glucose to glucose 1-phosphate and UMP and ADP-ribose to ribose 5-phosphate and AMP
	Digestion
FABP2	Fatty acid-binding protein intestinal
	Macrophages
APOC1	Activated when monocytes differentiate to macrophages positive regulation of cholesterol esterification
	Apoptosis/tissue regeneration
MSTO2P	Proliferation
LCMT1	Regulation of apoptotic process
PRDM11	Inhibits proliferation induces apoptosis
	Other
SPP2	Negative regulation of endopeptidase activity
SCGB1C1	Upper respiratory tract
RAMP1	Regulation of GPCR signaling pathway
S100G	Calcium ion/vitamin D binding mineral absorption
RANGRF	Positive regulation of GTPase activity
RPS28	Viral process
BEX4	
URAHP	
C19orf12	
FLJ37453	
TMEM141	
FXYD1	Positive regulation of sodium ion export
UNDEF425	
UNDEF464	

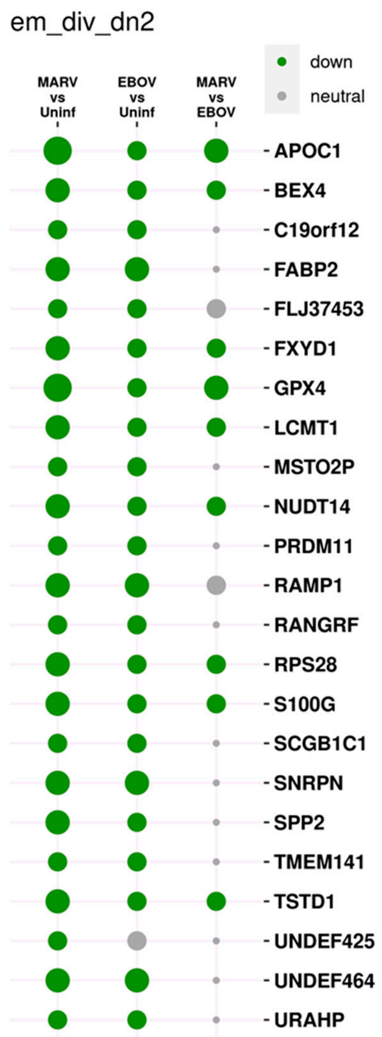

The balloon plot compares responses of genes to MARV-, EBOV- infected and uninfected samples against each other. The radius of circle is proportional to log2 (ratio), red is for positive, green is for negative values and gray is used when absolute values of log2 (ratio) < 0.6.

Table S5. Divergent pathways and genes upregulated by MARV but not EBOV infection: macrophages, complement, apoptosis, mitochondrial respiration, innate immunity, inflammation, digestion, T cells and the vascular system.

Gene	Process
	Macrophages
BPI	Negative regulation of IL-6 production expressed by Macrophages Bactericidal permeability-increasing protein
	Complement
CD46	Inactivates C3b and C4b protect host cell from damage by Complement innate immune response
	Apoptosis
XAF1	Response to interferon-beta proapoptotic
TNFRSF10A	Activation of NF-kB-inducing kinase activity cell apoptosis
BID	Positive regulation of apoptotic process

	Mitochondria/glycolysis/fatty-acid
SPR	Oxidoreductase activity and aldo-keto reductase (NADP) activity Nitric oxide biosynthetic process
ECHDC3	Fatty acid pathways(Macrophage M2)
	Innate immunity
IFI30	Antigen processing IFNG-mediated signaling pathway
TRIM22	IFNG-mediated signaling pathway antiviral ubiquitinates viral proteins
ICAM1	IFNG-mediated signaling pathway
	Inflammation
IL33	Positive regulation of inflammatory response
	Digestion
SULT2A1	Digestion Bile salt sulfotransferase
	T cells
CMTM6	Protects PD-L1 inhibits T cells
GZMH	Immune response T cell Granzyme H
	Vascular
EMP2	Positively regulates VEGF-A , integrin-mediated signaling pathway
RHOG	Platelet activation Rho-related
	Other
ARF1	Viral process ADP-ribosylation factor 1
RBM12B-AS1	RBM12B antisense RNA 1
RNF213	Protein ubiquitination E3 ubiquitin-protein ligase RNF213
UNDEF25	
CUNH17of100	
UNDEF113	

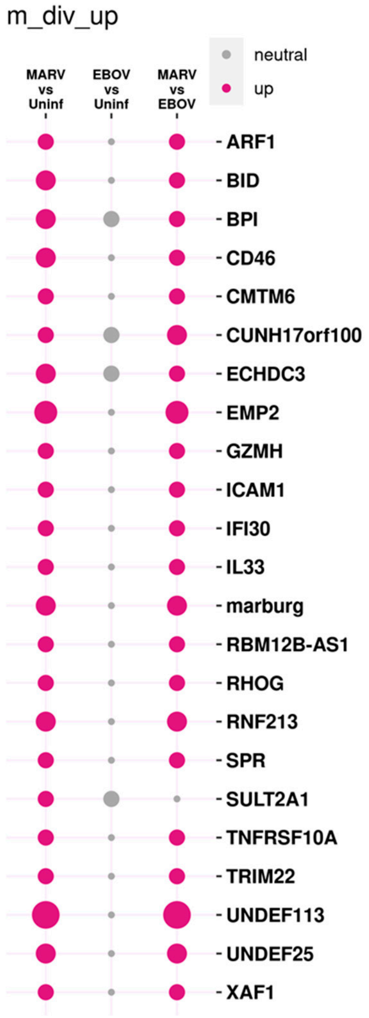

The balloon plot at the right compares responses of genes to MARV-, EBOV-infected and uninfected samples against each other. The radius of circle is proportional to log2 (ratio), red is for positive, green is for negative values and gray is used when absolute values of log2 (ratio) < 0.6.

Table S6. Divergent pathways and genes downregulated by MARV but not EBOV infection: mitochondrial, vascular, inflammation, digestion, innate immunity, complement, apoptosis and splicing.

Gene	Process
	Mitochondrial
MRPL41	Translation
NDUFV3	Mitochondrial electron transport NADH to ubiquinone
MDH2	Gluconeogenesis mitochondrial
TOMM6	Protein targeting to mitochondrion
NDUFB11	Respiratory electron transport chain
ATP5MPL	Mitochondrial membrane atp synthase
NDUFB1	Mitochondrial electron transport NADH to ubiquinone
NDUFA4	Mitochondrial electron transport NADH to ubiquinone
COA3	Positive regulation of mitochondrial translation

UQCRBP1	Ubiquinol-cytochrome c reductase binding protein pseudogene 1
	Inflammation/oxidative stress
NDUFB4	Response to oxidative stress
	Digestion
AMY2B	Digestion Alpha-amylase 2B
	Vascular
SERPINA4	Negative regulation of endopeptidase activity
ZNF24	Represses VEGF vascularization myelination
	Innate immune(complement)
C4BPB	Innate immune response
CCL16	Cellular response to interferon-gamma
RPS27A	Toll-like receptor signaling pathway
	Apoptosis/tissue regeneration
ATF4	Positive regulation of apoptotic process
JTB	Inhibits apoptosis induced by TGFB1
SIVA1	Viral entry into host cell apoptosis regulation
	Splicing
RBM8A	Regulation of alternative mRNA splicing via spliceosome
	Other
RPL4	Viral process
RPL18A	Viral process
RPL22	Viral process
RPLP1	Viral process
RPS17	Viral process
RPSAP58	Ribosomal small subunit assembly
RPP25L	Ribonuclease P protein subunit p25-like protein
CUNH6orf226	

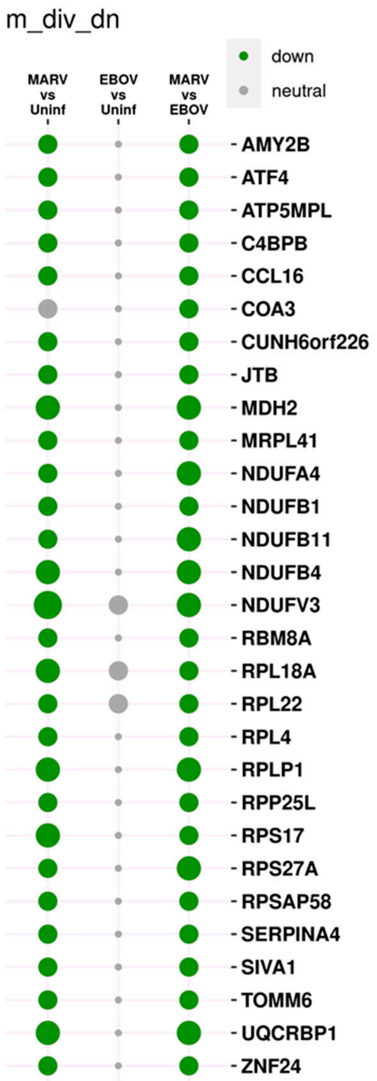

The balloon plot at the right compares responses of genes to MARV-, EBOV-infected and uninfected samples against each other. The radius of circle is proportional to log2 (ratio), red is for positive, green is for negative values and gray is used when absolute values of log2 (ratio) < 0.6.

Table S7. Divergent pathways and genes upregulated by EBOV but not MARV infection:.vascular function, inflammation, mitochondria, lipid metabolism, tissue regeneration.

Gene	Process
	Vascular
CYP11B2	Regulation of blood volume by renal aldosterone Cytochrome P450 11B2 mitochondrial
TMEM133/ARHGAP42	Inhibits RhoA activity to regulate vascular tone and control blood pressure
	Inflammation/stress
CYP11B1	Cortisol production stress response Immune response Cytochrome P450 11B1 mitochondrial
	Mitochondrial
MRPS33	Translation 28S ribosomal protein S33
PET100	Respiratory chain complex IV
NDUFA5	Respiration electron transport NADH to Ubiquinone
	Lipid/fatty-acid
ADIRF	Lipid metabolism
	Tissue regeneration
H19	lincRNA cell growth control
CENPW	Mitotic cell cycle
	Other
LINC00467	lincRNA 467
UNDEF312	

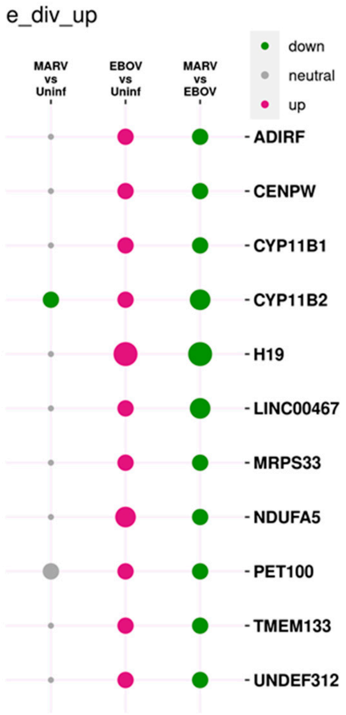

The balloon plot at the right compares responses of genes to MARV-, EBOV-infected and uninfected samples against each other. The radius of circle is proportional to log2 (ratio), red is for positive, green is for negative values and gray is used when absolute values of log2 (ratio) < 0.6.

Table S8. Divergent pathways and genes downregulated by EBOV but not MARV infection: innate immunity, coagulation and digestion.

Gene	Process
	Innate immunity
BST2	Response to IFNG
	Vascular
SERPINA13P	Protease inhibitor clotting
	Digestion
SLC51B	Bile secretion
	Other
UNDEF767	

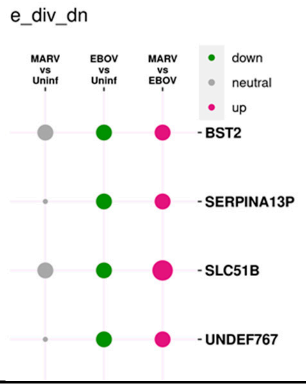

The balloon plot at the right compares responses of genes to MARV-, EBOV-infected and uninfected samples against each other. The radius of circle is proportional to log2 (ratio), red is for positive, green is for negative values and gray is used when absolute values of log2 (ratio) < 0.6.

Table S9. Correlations between various samples based on expression levels of genes.

**cb1 versus cb2 and cb3. Sample cb1 is dissimilar to cb2 and cb3**							
This study	cb2_Uninf	cb3_Uninf		ALL	cb2_Uninf	cb3_Uninf	
cb1_Uninf	0.77	0.74		cb1_Uninf	0.73	0.77	
**cb2 and cb3 are consistent with each other.**							
This study	cb2_Uninf	cb3_Uninf		ALL	cb2_Uninf	cb3_Uninf	
cb2_Uninf	1	0.94		cb2_Uninf	1	0.91	
cb3_Uninf	0.94	1		cb3_Uninf	0.91	1	
**Uninf versus MARV. cb1 is similar to MARV while cb2 and cb3 are not**							
This study	ab06_mb	ab07_mb	ab08_mb	ALL	ab06_mb	ab07_mb	ab08_mb
cb1_Uninf	0.74	0.86	0.71	cb1_Uninf	0.66	0.8	0.61
cb2_Uninf	0.26	0.46	0.24	cb2_Uninf	0.31	0.5	0.27
cb3_Uninf	0.39	0.56	0.37	cb3_Uninf	0.42	0.6	0.37
**Uninf versus EBOV. cb2/3 are similar to EBOV while cb1 is not**							
This study	ab01_eb	ab02_eb	ab03_eb	ALL	ab01_eb	ab02_eb	ab03_eb
cb1_Uninf	0.81	0.86	0.69	cb1_Uninf	0.82	0.85	0.58
cb2_Uninf	0.92	0.88	0.89	cb2_Uninf	0.88	0.86	0.75
cb3_Uninf	0.99	0.96	0.98	cb3_Uninf	0.96	0.93	0.79
**MARV versus MARV. The MARV samples are consistent with each other**							
This study	ab06_mb	ab07_mb	ab08_mb	ALL	ab06_mb	ab07_mb	ab08_mb
ab06_mb	1	0.97	1	ab06_mb	1	0.96	1
ab07_mb	0.97	1	0.96	ab07_mb	0.96	1	0.94
ab08_mb	1	0.96	1	ab08_mb	1	0.94	1
**EBOV versus MARV. The correlations between MARV and EBOV samples are low**							
This study	ab06_mb	ab07_mb	ab08_mb	ALL	ab06_mb	ab07_mb	ab08_mb
ab01_eb	0.47	0.64	0.45	ab01_eb	0.5	0.68	0.45
ab02_eb	0.6	0.75	0.58	ab02_eb	0.59	0.76	0.55
ab03_eb	0.38	0.54	0.36	ab03_eb	0.35	0.49	0.33
**EBOV versus EBOV. The EBOV samples are consistent with each other**							
This study	ab01_eb	ab02_eb	ab03_eb	ALL	ab01_eb	ab02_eb	ab03_eb
ab01_eb	1	0.99	0.96	ab01_eb	1	0.96	0.78
ab02_eb	0.99	1	0.94	ab02_eb	0.96	1	0.84
ab03_eb	0.96	0.94	1	ab03_eb	0.78	0.84	1

In each sub-table, on the left are the correlations between samples considering only the genes used in this study and on the right are correlations calculated using all the genes detected by mRNAseq. These are consistent with the hypothesis that MARV infection of ERBs elicits a strong reaction, while EBOV infection is more muted and similar to the uninfected samples. cb1 shows hallmarks of inflammation, justifying its exclusion from the study (also seen in Fig 3). Uninf = uninfected, mb = MARV-infected, eb = EBOV-infected. Sample names start with cb, or ab followed by numbers.
